# Updated Geriatric Cardiology Guidelines of the Brazilian Society of Cardiology - 2019

**DOI:** 10.5935/abc.20190086

**Published:** 2019-05

**Authors:** Gilson Soares Feitosa-Filho, José Maria Peixoto, José Elias Soares Pinheiro, Abrahão Afiune Neto, Afonso Luiz Tavares de Albuquerque, Álvaro César Cattani, Amit Nussbacher, Ana Amelia Camarano, Angela Hermínia Sichinels, Antonio Carlos Sobral Sousa, Aristóteles Comte de Alencar Filho, Claudia F. Gravina, Dario Celestino Sobral Filho, Eduardo Pitthan, Elisa Franco de Assis Costa, Elizabeth da Rosa Duarte, Elizabete Viana de Freitas, Emilio Hideyuki Moriguchi, Evandro Tinoco Mesquita, Fábio Fernandes, Gilson Soares Feitosa, Humberto Pierre, Ilnei Pereira Filho, Izo Helber, Jairo Lins Borges, Jéssica Myrian de Amorim Garcia, José Antonio Gordillo de Souza, José Carlos da Costa Zanon, Josmar de Castro Alves, Kalil Lays Mohallem, Laura Mariana de Siqueira Mendonça Chaves, Lídia Ana Zytynski Moura, Márcia Cristina Amélia da Silva, Maria Alice de Vilhena Toledo, Maria Elisa Lucena Sales de Melo Assunção, Mauricio Wajngarten, Mauro José Oliveira Gonçalves, Neuza Helena Moreira Lopes, Nezilour Lobato Rodrigues, Paulo Roberto Pereira Toscano, Pedro Rousseff, Ricardo Antonio Rosado Maia, Roberto Alexandre Franken, Roberto Dischinger Miranda, Roberto Gamarski, Ronaldo Fernandes Rosa, Silvio Carlos de Moraes Santos, Siulmara Cristina Galera, Stela Maris da Silva Grespan, Teresa Cristina Rogerio da Silva, William Antonio de Magalhães Esteves

**Affiliations:** 1 Escola Bahiana de Medicina e Saúde Pública, Salvador, BA - Brazil; 2 Universidade José do Rosário Vellano (UNIFENAS), Belo Horizonte, MG - Brazil; 3 Sociedade Brasileira de Geriatria e Gerontologia (SBGG), Rio de Janeiro, RJ - Brazil; 4 Universidade Federal de Goiás (UFG), Goiânia, GO - Brazil; 5 UniEVANGÉLICA, Anápolis, GO - Brazil; 6 Universidade de Pernambuco (UPE), Recife, PE - Brazil; 7 Hospital São Lucas, Pato Branco, PR - Brazil; 8 Universidade de São Paulo (USP), São Paulo, SP - Brazil; 9 Instituto de Pesquisa Econômica Aplicada (IPEA), Brasília, DF - Brazil; 10 Hospital São Julião, Campo Grande, MS - Brazil; 11 Universidade Federal de Sergipe (UFS), Aracaju, SE - Brazil; 12 Hospital São Lucas, Aracaju, SE - Brazil; 13 Universidade Federal do Amazonas (UFAM), Manaus, AM - Brazil; 14 Instituto Dante Pazzanese de Cardiologia, São Paulo, SP - Brazil; 15 Pronto-Socorro Cardiológico Universitário de Pernambuco (PROCAPE), Recife, PE - Brazil; 16 Universidade Federal da Fronteira Sul (UFFS), Chapecó, SC - Brazil; 17 Hospital Nossa Senhora da Conceição (HNSC), Tubarão, SC - Brazil; 18 Universidade do Estado do Rio de Janeiro (UERJ), Rio de Janeiro, RJ - Brazil; 19 Universidade Federal do Rio Grande do Sul (UFRS), Porto Alegre, RS - Brazil; 20 Universidade Federal Fluminense (UFF), Niterói, RJ - Brazil; 21 Instituto do Coração (Incor) da Faculdade de Medicina da Universidade de São Paulo (FMUSP), São Paulo, SP - Brazil; 22 Departamento de Insuficiência Cardíaca (DEIC) da Sociedade Brasileira de Cardiologia (SBC), Rio de Janeiro, RJ - Brazil; 23 Universidade Federal de São Paulo (UNIFESP), São Paulo, SP - Brazil; 24 Instituto de Cardiologia de Santa Catarina (ICSC), São José, SC - Brazil; 25 Hospital Agamenon Magalhães, Recife, PE - Brazil; 26 Sanofi, São Paulo, SP - Brazil; 27 Universidade Federal de Ouro Preto (UFOP), Ouro Preto, MG - Brazil; 28 Procardio Clínica Cardiológica, Natal, RN - Brazil; 29 Hospital Pró-Cardíaco, Rio de Janeiro, RJ - Brazil; 30 Pontifícia Universidade Católica do Paraná (PUC-PR), Curitiba, PR - Brazil; 31 Universidade de Brasília (UnB), Brasília, DF - Brazil; 32 Hospital Israelita Albert Einstein, São Paulo, SP - Brazil; 33 Hospital São Marcos, Teresina, PI - Brazil; 34 Hospital Universitário João de Barros Barreto, Belém, PA - Brazil; 35 Universidade do Estado do Pará (UEPA), Belém, PA - Brazil; 36 Hospital Madre Teresa, Belo Horizonte, MG - Brazil; 37 Universidade Federal da Paraíba (UFPB), João Pessoa, PB - Brazil; 38 Faculdade de Ciências Médicas da Santa Casa de São Paulo, São Paulo, SP - Brazil; 39 Universidade Federal do Rio de Janeiro, Rio de Janeiro, RJ - Brazil; 40 Instituto de Análises Clínicas de Santos (IACS), Santos, SP - Brazil; 41 Universidade de Fortaleza (UniFor), Fortaleza, CE - Brazil; 42 Hospital Vera Cruz, Belo Horizonte, MG - Brazil; 43 Hospital das Clínicas da Universidade Federal de Minas Gerais, Belo Horizonte, MG - Brazil; 44 Universidade de Itaúna, Itaúna, MG - Brazil

**Table:** 

Declaration of potential conflict of interest of authors/collaborators of Updated Geriatric Cardiology Guidelines of the Brazilian Society of Cardiology – 2019 If the last three years the author/developer of the Updated:							
Names Members of the Policy	Participated in clinical studies and/or experimental trials supported by pharmaceutical or equipment related to the guideline in question	Has spoken at events or activities sponsored by industry related to the guideline in question	It was (is) advisory board member or director of a pharmaceutical or equipment	Committees participated in completion of research sponsored by industry	Personal or institutional aid received from industry	Produced scientific papers in journals sponsored by industry	It shares the industry
Abrahão Afiune Neto	No	No	No	No	No	No	No
Afonso Luiz Tavares de Albuquerque	No	No	No	No	No	No	No
Álvaro César Cattani	No	No	No	No	No	No	No
Amit Nussbacher	No	No	No	No	No	No	No
Ana Amelia Camarano	No	No	No	No	No	No	No
Angela Hermínia Sichinels	No	No	No	No	No	No	No
Antonio Carlos Sobral Sousa	No	No	No	No	Bayer, Aché, Pfizer	No	No
Aristóteles Comte de Alencar Filho	No	No	No	No	No	No	No
Claudia F. Gravina	No	No	No	No	No	No	No
Dario Celestino Sobral Filho	No	No	No	No	No	Libbs	No
Eduardo Pitthan	No	No	No	No	No	No	No
Elisa Franco de Assis Costa	No	Abbott, Nutrition	No	No	No	Abbott, Nutrition	No
Elizabeth da Rosa Duarte	No	No	No	No	Torrent, Bayer, Ache, EMS	No	No
Elizabete Viana de Freitas	No	No	No	No	No	No	No
Emilio Hideyuki Moriguchi	No	No	No	No	No	No	No
Evandro Tinoco Mesquita	Novartis, Servier	No	No	No	No	No	No
Fábio Fernandes	No	No	No	No	Pfizer	No	No
Gilson Soares Feitosa	No	No	No	No	No	No	No
Gilson Soares Feitosa-Filho	No	No	No	No	No	No	No
Humberto Pierre	No	No	No	No	No	No	No
Ilnei Pereira Filho	No	No	No	No	No	No	No
Izo Helber	No	No	No	No	No	No	No
Jairo Lins Borges	No	LIBBS Farmacêutica	No	No	LIBBS Farmacêutica	No	No
Jéssica Myrian de Amorim Garcia	No	Pfizer	No	No	No	No	No
José Antonio Gordillo de Souza	No	No	Sanofi	No	No	No	No
José Carlos da Costa Zanon	No	No	No	No	No	No	No
José Elias Soares Pinheiro	No	No	No	No	No	No	No
José Maria Peixoto	No	No	No	No	No	No	No
Josmar de Castro Alves	No	No	No	No	No	No	No
Kalil Lays Mohallem	No	No	No	No	No	No	No
Laura Mariana de Siqueira Mendonça Chaves	No	No	No	No	No	No	No
Lídia Ana Zytynski Moura	No	No	No	No	No	No	No
Márcia Cristina Amélia da Silva	No	No	No	No	No	No	No
Maria Alice de Vilhena Toledo	No	No	No	No	No	No	No
Maria Elisa Lucena Sales de Melo Assunção	No	No	No	No	No	No	No
Mauricio Wajngarten	No	No	No	No	No	No	No
Mauro José Oliveira Gonçalves	No	No	No	No	No	No	No
Neuza Helena Moreira Lopes	No	No	No	No	No	No	No
Nezilour Lobato Rodrigues	No	No	No	No	No	No	No
Paulo Roberto Pereira Toscano	No	No	No	No	No	No	No
Pedro Rousseff	No	No	No	No	No	No	No
Ricardo Antonio Rosado Maia	No	No	No	No	No	No	No
Roberto Alexandre Franken	No	No	No	No	No	No	No
Roberto Dischinger Miranda	No	Aché, Bayer, Biolab, Hypera, Sanofi	Bayer, Boehringher, MSD	No	No	Biolab, Daiichi Sankyo, Pfizer	No
Roberto Gamarski	No	No	No	No	No	No	No
Ronaldo Fernandes Rosa	No	No	No	No	No	No	No
Silvio Carlos de Moraes Santos	No	No	No	No	No	No	No
Siulmara Cristina Galera	No	No	No	No	No	No	No
Stela Maris da Silva Grespan	No	No	No	No	No	No	No
Teresa Cristina Rogerio da Silva	No	No	No	No	No	No	No
William Antonio de Magalhães Esteves	No	No	No	No	No	No	No

## 1. General Aspects of Old Age, Risk Factors, and Prevention

### 1.1. Demographic and Epidemiological Aspects

Since the second half of the 20^th^ Century, survival has been democratized in numerous countries around the world. This means that more people are reaching more advanced ages. In Brazil, in 1980, of every 100 female live births, 30 could be expected to reach their 80^th^ birthday; in 2013, this number increased to 55. The average lifespan of the Brazilian population, consequently, increased nearly 12 years during this period. One of the factors responsible for this phenomenon was the decrease in advanced age mortality, which was the result of the control of theretofore lethal diseases. However, many of these diseases that ceased to be lethal are still not curable. As a consequence, the aged population, which continues aging and becomes more heterogeneous, has grown. This heterogeneity is due to differentiated gender, age, and epidemiological profile, among other factors. For example, of the approximately 26 million people age 60 or over, 56.4% were women, and 13.8% were age 80 or over. It is worth highlighting that not only is the aging process expected to increase, but the aged populated itself is also expected to age further. Or be it, the population age 80 or over is the one that grows the most, given the reduced mortality in this age group^1^ (Table 1). It is known that advanced age leads to the need to live with chronic, incapacitating diseases, which may compromise individual autonomy. In 2013, only 22.3% of elderly Brazilians declared that they had no chronic diseases. Approximately half, 48.6%, declared that they had 1 or 2 diseases, and 29.1% declared 3 or more. Women have a higher likelihood of contracting a disease than men, 81.2%, compared to 73.1%. This higher proportion of women in the elderly age group means a higher proportion of people with chronic morbidity^1^ (Table 2). Within diseases reported, cardiovascular diseases (CVD) are predominant. For example, 62.0% of men and 67.4% of women declare that they have hypertension, and 23.2% and 36.9% of men and women, respectively, declare high cholesterol. These diseases also constitute the main cause of death in the elderly population, accounting for 34.2% and 35.2% of deaths in men and women, respectively. Within CVD, acute myocardial infarction (AMI) and stroke stand out^2^ (Tables 3 and 4).

**Table 1:** Percentage distribution of the elderly population by sex and age^1^

	Men	Women	Total
60 to 69	56.5	56.3	56.4
70 to 79	30.7	29.4	30
80 to 89	10.8	12.2	11.6
90 or over	2	2.1	2
Total	100	100	100

Brazil, 2013.

**Table 2:** Proportion of elderly people with chronic diseases by number of pathological conditions^1^

	Men	Women	Total
None	26.9	18.8	22.3
1 to 2	49.4	48	48.6
3 or more	23.7	33.3	29.1

Brazil, 2013.

**Table 3:** Main causes of death in the elderly population by age^2^

	Men	Women
Circulatory system diseases	34.2	35.2
Neoplasm	19	15.5
Respiratory system diseases	14.3	14.7
Endocrine, nutritional, and metabolic diseases	6.5	8.9
Poorly defined	6.2	6.2
Others	19.9	19.5
Total	100	100
**		

Brazil, 2013.

**Table 4:** Main causes of death due to circulatory system disease by sex^2^

	Men	Women
Acute myocardial infarction	26	21.4
Strokes not specified as hemorrhagic or ischemic	13.7	13.7
Heart failure	8.2	9.4
Others	52	55.5
Total	100	100

Brazil, 2013.

This indicates a greater need for prevention, with lifestyle changes, alcohol and tobacco control, better diet, and physical exercise being able to contribute to a reduction in CVD. In summary, it is possible to affirm that humanity seems to be making the dream of long life come true, but it is necessary to avoid the Tithonus trap. Tithonus was a mythical Trojan hero who was granted eternal life; he forgot, however, to ask for eternal youth. Eventually he was transformed into a cricket. Ulysses, on the other hand, declined the gift of immortality, Ulysses, on the other hand, declined the gift of immortality, preferred remain owner of his destiny and his soul (Homero). Or be it, living a long life, with autonomy, should be humanity's dream.

### 1.2. Interpretation of Frailty

Frailty is a biological syndrome characterized by decreased homeostatic reserve and resistance to various stressors. It results in cumulative decreases in multiple physiological systems and leads to increased vulnerability and unfavorable clinical outcomes, such as falls, impaired mobility and functional decline, hospitalization, institutionalization, and a higher risk of death.^3^ This state of vulnerability causes an apparently minor injury (e.g., infection, introduction of a new medication, or even a small surgery) to lead to an evident, disproportional change in the patient's state of health; these changes may by exemplified as alterations from independent to dependent status, from able to move to immobile, from balance and stable gait speed to risk of falling, or from lucid to delirious.^4,5^

There is an overlap, but not a concurrence in the incidence of frailty, incapacity, and multimorbidity (coexistence of two or more chronic diseases). Although they are less frequent, there are frail individuals who have neither incapacity nor multimorbidity.^4^ Sarcopenia (decreased muscle mass and function) is a component of the syndrome of frailty, which is more multifaceted and complex than sarcopenia alone.^5^

Clinical presentation results not only from a single well defined disease, but rather from the accumulation of impairments in multiple organic systems, and it occurs when the accumulated effects of these impairments compromises the organism's compensatory capacity. A systematic review demonstrated that the prevalence of frailty among community-dwelling elderly people was 10.7% (varying from 4.0% to 59.1%).^6^

In CVD patients, frailty confers a 2-fold risk of death, and this effect continues after adjusting for comorbidities and age. Numerous studies have also demonstrated an increase in the prevalence of frailty among patients with CVD, such as coronary artery disease (CAD), heart failure (HF), heart valve disease, etc. A higher risk of complications and mortality has also been identified in frail elderly patients who undergo cardiovascular interventions such as surgery and angioplasty.^7^

Frailty may potentially be prevented or treated, and many studies have demonstrated that exercise, protein/caloric supplementation, vitamin D supplementation, and reduction and optimization of polypharmacy may decrease levels of frailty, thus minimizing adverse outcomes and risks of interventions.^5,8^

The identification of frail elderly patients is advocated so that multidimensional interventions may be implemented, mainly physical and nutritional rehabilitation, which reduces or postpones adverse outcomes and provides risk prognosis. It is necessary to emphasize that the identification of frailty does not need to be seen as a reason to exclude or suspend treatment, but rather as a means of programming individualized, patient-centered interventions.^5,7^

Fried et al. (2001), in a longitudinal cardiovascular cohort study, identified the following manifestations for this syndrome: unintentional weight loss, muscular weakness, exhaustion (fatigue), decreased gait speed, and decreased degree of physical activity. Based on this, they proposed diagnostic criteria known as the "Fried et al. Frailty Phenotype",^3^ or "Cardiovascular Health Study Frailty Screening Scale".^3,5^ These criteria have been criticized, insofar as those referring to exhaustion and decreased physical activity are not objective and are difficult to evaluate in daily practice with elderly patients. Other indexes and scales for diagnosis have been proposed, such as Rockwood Clinical Frailty Scale,^9^ the Gérontopôle Frailty Screening Tool,^10^ the FRAIL scale proposed by Van Kan and Morley,^11^ the Groningen Frailty Indicator,^12^ the Tilburg Frailty Indicator,^13^ the PRISMA-7 questionnaire,^14^ the VES-13 Scale,^15^ and the Edmonton Frailty Scale.^16^ The latter five instruments have been transculturally adapted and/or validated in Brazil. Studies have demonstrated that the 5-meter gait speed test is a useful tool for evaluating frailty in elderly patients referred for percutaneous aortic valve implantation.^17,18^ The incorporation of this tool into the Society of Thoracic Surgeons (STS) score improved its ability to predict adverse events. For a given STS score, the risk of mortality or morbidity was 2-3 times greater in patients with slow gait speed.^17,18^ Regardless of the instrument used to screen and identify, the syndrome of frailty should be investigated in all individuals over age 70 and in elderly patients with CVD, even if they are below this age group, and prevention and treatment measures should be put into practice.^5,7,8^

### 1.3. Particularities in the Evaluation of Elderly Patients

Aging is a risk factor for most CVD, as well as numerous comorbidities, making the elderly the most heterogeneous and most complex adult age group.^19^ Generally speaking, the healthcare system is poorly prepared to attend patients with multimorbidities, given that they require greater individualization, as well as assistance from a multiprofessional team that works integratedly.^20,21^

Interventions which are clearly beneficial in adults are, generally, also beneficial for elderly patients. However, the peculiarities which exist regarding evaluation of elderly patients are fundamental for their individual treatment. The evaluation of elderly patients should be performed using the Broad Geriatric Assessment (*Avaliação Geriátrica Ampla*, AGA).^22^ This is a multidimensional, generally interdisciplinary, diagnostic process for determining impairments, inabilities, and disadvantages in elderly patients and, thus, planning their medium- to long-term care and assistance. The AGA prioritizes functional status and quality of life, facilitating communication between interdisciplinary team members. It should be applied to frail elderly patients and patients with multimorbidities. The AGA is also an important predictor of unfavorable outcomes, i.e., it has prognostic value for surgery, oncology, and orthopedic patients.^22^ The AGA is fundamental in the context of evaluating elderly patients. It includes, at least, 4 principal dimensions, which are functional capacity, medical conditions, social functionality, and mental health.^22^

Independent elderly patients with a long life expectancy should be treated comprehensively in a manner that combines prevention and intervention. On the other hand, pre-frail and frail patients require more attention regarding their individual needs and priorities, as well as risk-benefit assessment for individualized therapeutic decisions.^19,20^ Goals to be reached should, equally, depend on functional status, without contraindicating any treatment whatsoever exclusively on account of age.

Considering the high prevalence of multimorbidities and the high evolution of therapeutic options, polypharmacy has become very frequent in elderly patients, posing further challenges to case management.^19,20^ Understanding the advantages and disadvantages of every treatment is fundamental to adequate elderly treatment. This may only be scaled through the AGA. Familiarity with the AGA is, thus, essential to the evaluation and introduction of a determined treatment in an elderly patient.^21,22^

### 1.4. Particularities in the Treatment of Elderly Patients

In treating elderly patients, priority is given to the patient who is ill, rather than to the illness, and to controlling the disease, rather than curing it. It is essential to know the disease, the patient who has the disease, and the treatment. CVD is frequent, and, even when there are few manifestations, it brings increased risks; elderly patients with diseases present comorbidities and high biopsychosocial vulnerability; treatments are more susceptible to undesired effects. Thus, evaluation of multiple clinical and psychosocial domains is fundamental. Owing to the fact that evidence is often lacking, conduct should be individualized. Decisions should be shared, and it is necessary to consider risk-benefit ratio and life expectancy. In elderly patients, treatment indication requires more caution. Although therapeutic goals are less precise, excluding them solely on the basis of age implies omission.^23-26^

Orientations regarding lifestyle changes are recommended, as in younger age groups. This may, however, cause undesired effects, especially if the changes are misunderstood or misapplied. Changing old habits requires attention.

Pharmacological treatments should: prioritize conditions and restrict number of medications, simplify posology, evaluate and stimulate satisfactory adherence even in secondary prevention following AMI,^26^ provide orientation regarding problems related to self-medication, consider modifications in pharmacology related to age which, generally, recommend reducing doses, and evaluate possible drug interactions, given that "polypharmacy" is common. Beers Criteria, informally known as the "Beers List," are a reference on safety in prescribing medications to the elderly. They were created in 1991, by the geriatrician Mark H. Beers, and they are periodically revised, the 2015 version being the most recent.^27^

In the United States, more than one third of emergency room visits due to adverse effects of substances occur in individuals over age 65. They imply hospitalization of more than 40% of cases, and this frequency is increasing over time. Of these visits, nearly 60% were related to the use of anticoagulants, antidiabetic agents, and opioid analgesics, and nearly 2% were related to restricted use medications, in accordance with the Beers Criteria.^28^

Recently, the Food and Drug Administration Adverse Event Reporting System (FAERS) drew attention to evaluating the eventual need for regulatory action for the following: the anticoagulants apixaban, edoxaban, rivaroxaban, and dabigatran, due to reports of vasculitis; ivabradine, due to potential signs of ventricular arrhythmias; and midodrine, due to reports of interactions with monoamine oxidase inhibitors (MAOI) which could trigger a stroke.^29^

Interventional treatments should be carefully based on criteria, with the participation of heart teams, and performed by experienced and qualified teams, given that they present more frequent and severe complications.

A noteworthy example of this scenario is the need for hospitalization and admission to skilled nursing facilities in 4 of every 5 elderly patients who received an implantable cardioverter-defibrillator for secondary prevention of sudden cardiac death, even though they survived at least 2 years.^30^

### 1.5. Diabetes Mellitus in Elderly Patients

The National Health Survey conducted by the Brazilian Institute of Geography and Statistics (IBGE, 2013) showed a 19.9% prevalence of diabetes mellitus in individuals in the 65-74 age group.^1^ In diabetic adults, there is an increase in mortality and a decrease in functional capacity with consequent increase in the risk of institutionalization.^31^ The presence of multimorbidities and comorbidities associated with this group's high heterogeneity means that the elderly are often excluded from randomized clinical trials, making disease management more difficult in this population.^31,32^

Diagnostic criteria for diabetes mellitus in the elderly are similar to those in younger populations: (1) fasting blood glucose ≥ 126 mg/dL; or (2) random blood glucose ≥ 200 mg/dL, associated with disease symptoms; or (3) blood glucose 2 hours after a 75-g glucose load ≥ 200 mg/dL; or (4) glycated hemoglobin (HbA1C) ≥ 6.5% (provided that the laboratory is standardized). The American Diabetes Association (ADA) recommends that individuals who are overweight as a risk factor and all adults ≥ age 45 be screened for diabetes every 1 to 3 years, with fasting blood glucose, glycated hemoglobin dosage, or oral glucose tolerance test, for the benefit of early diagnosis, early treatment, and prevention of complications.^31^

Elderly individuals with diabetes are at a higher risk of developing geriatric syndromes, such as polypharmacy, cognitive impairment, urinary incontinence, falls, and chronic pain. When individuals with these syndromes develop diabetes, their clinical condition worsens. Thus, in addition to screening for complications, multidimensional evaluation of elderly diabetic individuals is also fundamental. It becomes imperative to perform AGA with mental, functional, nutritional, and social evaluations for these individuals in order to define goals to be met for each patient.^32^ The objective should be defined between two options: intensive blood glucose control, with less progression of chronic complications; or standard blood glucose control, in order to avoid only symptoms of hyperglycemia and acute complications.

The United Kingdom Prospective Diabetes Study (UKPDS), although it excluded elderly patients, showed the benefits of intensive blood glucose control in individuals as they age, with posterior follow-up.^33,34^ There are 3 main randomized clinical trials with the participation of elderly patients and intensive blood glucose control. The Action to Control Cardiovascular Risk in Diabetes (ACCORD) study was interrupted due to mortality in the youngest group; however, hypoglycemia and other adverse effects of treatment were more common in elderly patients;^35,36^ in the Action in Diabetes and Vascular Disease: Preteraax and Diamicron MR Controlled Evaluation (ADVANCE) study, the risk of hypoglycemia and hospitalization increased significantly;^37^ and in the Veterans Affairs Diabetes Trial (VADT) study, there were no benefits, with the exception of decreased progression of microalbuminuria.^38^ Two retrospective studies (U.K. General Practice Research Database, 2009^39^ and The Diabetes and Aging Study, 2011) show a U-shaped curve relating mortality and blood glucose levels.^40^ Individualization of treatment is, thus, imperative in elderly patients in accordance with their clinical, functional, and life expectancy profile, as demonstrated in Table 5, with treatment goals for arterial hypertension and dyslipidemia in elderly patients with diabetes.

**Table 5:** Treatment goals regarding blood glucose, and dyslipidemia in elderly patients with diabetes

Patient characteristics/health status	Rationale	Reasonable HbA1C goal	Fasting or preprandial blood glucose (mg/dL)	Bedtime blood glucose (mg/dL)	Blood pressure (mmHg)	Lipids
Healthy (few coexisting chronic diseases, cognitive and functional state intact)	Long life expectancy	< 7.5%	90–130	90–150	< 140/90	Statin, provided there is no contraindication or intolerance
Complex/intermediate (multiple coexisting chronic diseases, impaired IADL, or mild to moderate cognitive impairment)	Intermediate life expectancy, high treatment burden, vulnerability to hypoglycemia, fall risk	< 8.0%	90–150	100–180	< 140/90	Statin, provided there is no contraindication or intolerance
Very complex/ poor health (long-term care or end-stage chronic disease, moderate to severe cognitive impairment, or 2+ BADL dependencies)	Limited life expectancy makes benefit uncertain	< 8.5%	100–180	110–200	< 150/90	Consider the probability of benefits of statin (secondary prevention, rather than primary)

BADL: basic activities of daily living; HbA1C: glycosylated hemoglobin; IADL: instrumental activities of daily living.

Source: American Diabetes Association. Older adults. Diabetes Care. 2017; 40 (suppl.1):S99-S104.^32^

### 1.6. Tobacco Use

The influence of tobacco use in elderly individuals occurs due to anatomical and physiological alterations in a cumulative process which leads to endothelial dysfunction, increased platelet adhesion, decreased high-density lipoprotein cholesterol (HDL-c), and increased low-density lipoprotein cholesterol (LDL-c), among other alterations.^41^ Tobacco use is common in the elderly population, and it is an important cause of morbidity and mortality, including CVD, peripheral vascular disease, cerebrovascular disease, cancer, and obstructive pulmonary disease. On the other hand, the tobacco cessation has benefits, even in elderly patients, with respect to the prevention of these diseases or, at least, to slowing the decline of pulmonary function.^42^ The Systolic Hypertension in the Elderly Program Study^43^ observed patients with an average age of 72 and showed a significant increase in AMI, sudden death, and stroke in smokers, compared with non-smokers. Exposure to long periods of passive tobacco use increases the risk of developing CAD. Kawachi et al. (1997)^44^ followed 32,000 non-smoking women, between the ages of 36 and 71, for 10 years, and found that the relative risk of developing coronary heart disease increased in women exposed to smoking. Occasional exposure to cigarettes increased their relative risk by 1.58, and regular exposure increased the relative risk by 1.91.^45^ Tobacco use constitutes a risk factor for dementia, and cessation may reduce the burden of dementia. Passive exposure to smoking may also increase the risk of dementia.^46^ Studies show that elderly smokers have a lower intention of quitting in comparison with younger smokers; they have, on the other hand, a higher likelihood of success when they do try to stop smoking.^47,48^ Success in stopping is frequently achieved after an acute coronary event, aggravation of chronic obstructive pulmonary disease (COPD), or symptomatic and limiting peripheral arterial disease. Medical advice to cease smoking should be firm, with emphasis placed on the short- and medium-term benefits. Aggressive practices related to tobacco cessation should be adopted.^49,50^ Evidence shows the efficiency of using the 4 As method in elderly patients, namely: ask, advise, assist, and arrange follow up.^51,52^ Different approaches, such as interventions through individual counseling performed by healthcare professionals, age-appropriate self-help material, use of nicotine (transdermal patches or chewing gum), or use of specific medications, e.g. bupropion), have also been shown to be efficient in treating tobacco use.^53-55^

**Table:** 

Recommendations	Grade of recommendation	Level of evidence
Tobacco use is a modifiable risk factor for CVD in elderly individuals and cessation is recommended	I	C
Use of multidisciplinary approaches, with the 4 As Method, is recommended: ask, advise, assist, and arrange follow up	I	C
Nicotine/bupropion transdermal patches or chewing gum may be used to cease tobacco use	IIa	C

CVD: cardiovascular disease.

### 1.7. Obesity

The prevalence of overweight status and obesity has increased over the past decades in all age groups, including the elderly.^56,57^

Both obesity and overweight status have been associated with the risk of all cause and CVD mortality, in the general population.^58-60^

The majority of these studies mainly involved young adult patients, making this relationship less evident in the elderly.^61-64^

Some meta-analysis studies have reported that overweight and obese elderly individuals, when compared with elderly individuals within the normal weight range, had lower mortality rates and lower or no risk of CVD. This effect has been called the "obesity paradox".^65-67^

In addition to possible confounding factors in these studies, other reasons may be involved. The index used to measure and classify body mass was the body mass index (BMI). Degrees of obesity adopted by the World Health Organization (WHO), with respect to BMI, are: overweight (25.0 to 29.9 kg/m^2^) and obese (over 30.0 kg/m^2^).^68^ Variables such as age, sex, and race may affect BMI. With aging, changes in body composition occur, such as increased visceral fat and decreased muscle mass. Loss of height may also occur, owing to compression of vertebral bodies or kyphosis. In this manner, BMI becomes less precise in measuring fat mass. When used alone, it is not able to be an accurate predictor of CVD risk in elderly patients. For instance, some elderly individuals may be considered overweight by body fat patterns without having a BMI over 25 kg/m^2^.

Using BMI alone, we may be underestimating the degree of adiposity in individuals who lost muscle mass. Central obesity and nutrition are factors which seem more important in relation to mortality and CVD risk in this population. Some authors suggest that waist circumference (WC) could be a particularly important measure for elderly patients, which would be better than BMI at evaluating risk, given its association with visceral adiposity.^69,70^

Another study indicates that the presence or absence of metabolic syndrome is more important than BMI in obese elderly patients, thus dividing this population into "healthy obese" (without metabolic syndrome) and obese with metabolic syndrome. The latter group has been strongly associated with increased risk of CVD regardless of BMI.^71^

More studies are necessary to clarify the interrelation between aging, obesity, and cardiovascular risk and what the best measures parameter(s) would be. Weight management in the elderly and efforts to promote healthy aging should be based on an individual approach, taking into consideration the maintenance of muscle mass and strength, comorbidities, functional and social status, physical activity, and quality of life. Intentional weight loss in obese elderly patients improves their cardiovascular risk profile, reduces chronic inflammation, and is correlated with improved quality of life. Unintentional weight loss requires careful clinical assessment of the underlying cause. Furthermore, the identification of elderly patients with sarcopenic obesity is relevant to prognosis. Sarcopenia and sarcopenic obesity have been associated with a higher risk of CVD, especially in elderly men with this type of obesity.

### 1.8. Sedentarism

Regular physical activity is essential to healthy aging. Considering that aging in inevitable, the rhythm and magnitude of decline in physiological function may be influenced by an intervention comprising exercise/physical activity (Table 6).^72,73^

**Table 6:** Centers for Disease Control and Prevention exercise guidelines for adults over age 65

Substantial health benefits	2 hours and 30 minutes (150 minutes) of moderate-intensity aerobic activity per week Muscle strengthening activities 2 or more days per week 1 hour and 15 minutes (75 minutes) of vigorous-intensity aerobic activity
Additional health benefits	5 hours (300 minutes) of moderate-intensity aerobic activity per week Muscle strengthening activities 2 or more days per week 2 hours and 30 minutes (150 minutes) of vigorous-intensity aerobic activity

Adapted from: Centers for Disease Control and Prevention (CDC). Physical activities for older adults. Available at: www.cdc.gov/features/activity-older-adults/index.html. Accessed: 18/02/2016.

Aging is associated with skeletal muscle mass loss; reduced muscle strength, flexibility, cardiac output, and pulmonary function; changes in hormonal and immune system regulation; reduced bone density, and higher prevalence and incidence of sedentarism.^74^

In sedentary elderly patients, walking may be a practical solution, evaluating heart rate (HR) before and after exercise. It is necessary to recognize that elderly people do not represent a uniform group of patients and chronological age in itself does not identify this special group.^75^

Sedentarism is an important risk factor for CAD in elderly individuals. Some studies demonstrate that the relative risk of CAD attributable to sedentarism is comparable to that of hypertension, hyperlipidemia, and tobacco use. Sedentarism, as an important risk factor, is, in most cases, directly or indirectly associated with the causes or aggravation of various diseases, such as obesity, diabetes, arterial hypertension, anxiety, depression, dyslipidemia, atherosclerosis, respiratory disease, osteoporosis, and cancer.^76,77^ Systematic physical exercise helps control systemic arterial hypertension (SAH), by reducing peripheral arterial resistance, increasing HDL-c, reducing obesity and triglycerides, improving blood glucose control, preventing coronary disease, and decreasing mortality.^77,78^

Furthermore, it improves sleep quality, cognitive function, and short-term memory; decreases degree of depression; reduces or slows the onset of dementia; reduces the risk of colon, breast, prostate, and rectal cancer; increases bone density; and decreases the incidence of femur and vertebrae fractures.^77^

In elderly patients, pre-exercise clinical evaluation is very important. The goal of exercise and cardiovascular rehabilitation in elderly patients is to improve their functional capacity as much as possible. These objectives are reached through programs that aim to increase aerobic capacity, muscle strength, and flexibility.^72,79-82^

The amount of physical activity should be individualized, considering each patient's comorbidities and peculiarities.^73,74,79^

Elderly individuals should spend more time warming up before and cooling down after activity. The warm-up phase includes flexibility and movement exercises, which facilitate musculoskeletal biomechanics. The post-exercise cool-down phase allows for the gradual dissipation of body heat and consequent peripheral vasodilatation. Musculoskeletal injuries may be decreased by avoiding high impact activities, such as running and jumping. Extreme care is necessary for activities using free weights, given the risk of accidents, especially in less skilled or more frail elderly patients.^72,80^ Walking briskly is an excellent way to obtain physical conditioning, with gradual increases in pace and distance covered.^81^ Elderly patients should be instructed to reduce exercise intensity on humid or hot days, given that skin blood flow decreases with age, consequently lowering the efficiency of sweating and thermal regulation.^77^ Practicing resistance exercise twice weekly is also recommended.

Pre-participation assessment should begin with patient history and clinical exam, focusing on the particularities of this population, which often has silent atherosclerotic disease. Complementary investigation should be oriented by clinical data, avoiding high costs, which are sometimes prohibitive and may discourage physical exercise. Resting electrocardiogram (EKG) for the elderly has limited application as a pre-selection exam for physical activity.

If possible, an exercise test (ET) should be performed in all elderly patients before initiating physical activity. The prevalence of coronary disease increases with age; the rationale behind the ET in this population may, thus, be even greater than in the general adult population.^72,79,80^ The ET is a procedure during which the patient undergoes programmed and individualized exercise, with the aim of evaluating clinical, metabolic, hemodynamic, autonomic, electrocardiographic, and, eventually, ventilatory responses to exercise. In elderly patients, modified protocols are used to perform the ET.^79^ If there are contraindications to performing the ET, stress EKG or scintigraphy should be performed. A Holter monitor is used to stratify risk in elderly patients with arrhythmias detected during EKG or ET, as well as those with a history of syncope.^72,82^

Adherence to physical activity in this group has been increasingly positive. It is always necessary to consider that an active or latent pathological process may by present in an elderly individual and that the ET may contribute to defining it.^83,84^

**Table:** 

Recommendations	Grade of recommendation	Level of evidence
Clinical exam and electrocardiogram	I	C
Electrocardiogram, exercise test, or myocardial scintigraphy in medium-risk patients or in moderate to intense exercise	IIa	C
Physical exercise	I	A
Resistance exercise	IIa	C

### 1.9. Dyslipidemia in Elderly Patients

Dyslipidemia is a frequent diagnosis in elderly patients, mainly in women, owing to the fact that LDL-c levels tend to rise as they advance in age, especially after menopause; in men, however, LDL-c tends to decrease after age 55. Unlike in young adults, cases of de novo dyslipidemia are rare, and cases of dyslipidemia secondary to hypothyroidism (especially in women), diabetes mellitus, glucose intolerance, nephrotic syndrome, obesity, alcoholism, or use of medications such as thiazide diuretics and non-selective beta-adrenergic receptor blockers, are more common.^85^

In relation to treatment, as elderly patients are often already at high risks (owing to the factor of age), the approach to dyslipidemia, regarding therapeutic decisions, should give greater consideration to the patient's good general and mental state, his or her socio-economic conditions, family support, comorbidities present, and the use of other drugs that may influence adherence to and maintenance of therapy. Non-pharmacological orientations should follow the same principals of indication for young adults, more carefully observing caloric, protein, and vitamin intake needs and physical conditions for practicing exercise (recommendation I, evidence B). It is necessary to reiterate the importance of ceasing habits of smoking and excessive consumption of alcoholic beverages. After 90 days, if there is no response, drug treatment may be initiated, with the following precautions: (1) always start with low doses and, if necessary, increase, progressively; (2) analyze the cost-benefit ratio; and (3) verify the existence of socioeconomic conditions for maintaining long-term treatment and performing periodical clinical and laboratory exams, due to the higher likelihood of collateral effects and drug interactions.^85^

For hypercholesterolemia, statins are the first choice.^86^ Tolerance is good; there is not a high incidence of undesired effects, even though muscle pain, cramps, and weakness, which are sometimes confounded with osteomuscular disease, may occur, even in low doses. Evidence from subgroup analyses in primary and secondary prevention studies and the Pravastatin in Elderly Individuals at Risk of Vascular Disease (PROSPER) study,^87^ specially designed for elderly patients with or without previous manifestations of atherosclerosis, demonstrated the following benefits to treatment for this age group: reduction of coronary events (grade of recommendation IIa, level of evidence B), stroke (grade of recommendation IIa, level of evidence B), and preservation of cognitive functions (grade of recommendation IIb, level of evidence B). When maximum statin dosages are not sufficient to meet recommended LDL-c goals, ezetimibe may be associated with the statins (grade of recommendation IIb, level of evidence B).^88^

In cases of hypertriglyceridemia, fibrates are used (provided there are no gallstones or renal insufficiency). Fibrates and statins may be associated in cases of mixed dyslipidemia (elevated LDL-c and triglycerides), mainly with reduced HDL-c (grade of recommendation IIb, level of evidence D).^88^

In secondary dyslipidemias, the fundamental concern is treating the triggering disease and substituting or removing inductor drugs. We should remember that elderly individuals, generally, use other drugs metabolized by cytochrome P450 (CYP450), which have the possibility of interacting with lipid-lowering agents, thus altering their blood concentration (grade of recommendation IIb, level of evidence D).^88^

### 1.10. Depression and Cardiovascular Disease

Depression and anxiety are highly prevalent in individuals with CAD and other CVD. They have been also been considered independent risk factors for CAD and CVD, in addition to altering their natural history.^89,90^

Depression is disproportionally more frequent among CAD patients, with prevalence between 20% and 40%. It has also been reported that depression is prospectively associated with an increased risk of developing CAD,^91,92^ including AMI,^93^ at some point during life, as well as an increased risk of mortality.^94^ A 60-month follow-up study of 158 patients who suffered AMI revealed that greater depression was a significant predictor of mortality and adverse cardiac events.^95^

Collateral effects of antidepressants on the cardiovascular system have been reported. These include bradycardia, tachycardia, hypertension, hypotension, orthostatic hypotension (OH), EKG alterations, altered electrolytes, reduced cardiac conduction, arrhythmias, and sudden cardiac death.^96^

#### 1.10.1. Treating Depression and Anxiety in Patients with Cardiovascular Disease

First generation antidepressants include MAOI and tricyclic and tetracyclic antidepressants (TCA and TeCA, respectively); second-generation antidepressants include selective serotonin reuptake inhibitors (SSRI), selective norepinephrine reuptake inhibitors (SNRI), and atypical antidepressants.^89,96^

Even though MAOI (phenelzine, tranylcypromine, moclobemide, selegiline, etc.) are effective, they present several unfavorable collateral effects, mainly OH, tachycardia, and hypertensive crises; the latter are also associated with stroke and acute aortic dissection and should, thus, be avoided in patients with CAD.^89,96^

The cardiovascular collateral effects of TCA (imipramine, amitriptyline, nortriptyline, desipramine, clomipramine, doxepin, maprotiline, etc.) are fairly well known, namely, increased HR, OH, slowed cardiac conduction, and increased QT interval variability.^89,97^ These effects, which have been reported not only in patients with CVD but also in people without previous cardiac disease, in addition to their anticholinergic action, make this class of drugs inappropriate for treating depression in elderly patients.^96^

SSRI (fluoxetine, sertraline, paroxetine, citalopram, escitalopram, fluvoxamine, etc.) are considered the medications of choice for treating depression and anxiety in most cases, due to their acceptable safety profile and higher margins of non-toxic levels in comparison with other classes of antidepressants.^89,96^

Regarding efficacy of SSRI in decreasing symptoms of depression, all meta-analyses of selected indicators have shown that these antidepressants are more effective than placebo.^98^

SSRI may cause prolonged QT intervals (reported mainly with fluoxetine and citalopram), but they do not generally lead to life-threatening arrhythmias in therapeutic doses. Citalopram appears to be the most cardiotoxic SSRI (conduction disturbances and arrhythmias).^96^

Most causes of prolonged QT interval and subsequent torsade de pointes (TdP) induced by SSRI are observed in patients with underlying vulnerabilities, such as congenital long QT syndrome, recent AMI, hypokalemia, or hypomagnesemia, or in cases of substance overdoses.^96^

Within this class, there is some evidence that escitalopram and sertraline have the best balance between effectiveness and acceptability for pharmacological treatment of depression in cardiac patients.^99^

In summary, SSRI probably do not cause adverse effects when used according to the recommended dosages, and it has been suggested that, through complex mechanisms, they may bring some benefits to the cardiovascular system, such as lower rates of AMI in comparison with other types of antidepressants, especially TCA.^96^

As there are still no robust clinical orientations, patient treatments should be individualized in relation to potential risks and benefits. Additional studies are necessary to verify the exact cardiovascular safety profile.^96^

Regarding selective serotonin and norepinephrine reuptake inhibitors (SSNRI) (venlafaxine, desvenlafaxine, reboxetine, duloxetine, etc.), venlafaxine is associated with severe cardiotoxicity, only when given in high doses. Left ventricular (LV) failure, even in patients with no prior history of CVD, has also been reported in the literature.^89,96^ It is recommended to monitor blood pressure (BP) in patients who take SSNRI (especially venlafaxine), given that it has been reported to increase in epidemiological studies.^96^

Regarding atypical antidepressants (mirtazapine, agomelatine, nefazodone, trazodone, etc.), mirtazapine, in high doses, may cause hypotension and affect HR. Trazodone has minimal cholinergic activity; it may cause OH, and, in excess, prolonged QT and slowed atrioventricular conduction.^96^

In addition to pharmacological treatment, psychotherapy and the prescription of non-medical treatments, such as physical activity, especially aerobic exercise and cardiac rehabilitation, are also indicated. These improve prognosis and patient quality of life and reduce risks of evolution of CAD and CVD.^89,99^

### 1.11. Other Cardiovascular Risk Factors

Traditional risk factors explain only half of CVD cases, which present high morbimortality rates. Several studies have been developed to look for possible new risk factors, known as emerging risk factors, as well as means of early diagnosis of CVD by investigating signs of subclinical atherosclerosis. The emerging risk factors covered in these Guidelines are hyperuricemia, C-reactive protein (CRP), vitamin D, genetic factors, coronary calcium score (CCS), and investigation of subclinical atherosclerosis.

#### 1.11.1. Hyperuricemia

Recent epidemiological studies have demonstrated that hyperuricemia is frequently observed in patients with CVD or high risks thereof, such as arterial hypertension, CAD, peripheral vascular disease, HF, and stroke.^100^

A recent meta-analysis of observational prospective studies on hyperuricemia and risk of stroke demonstrated a significant increase in the risk of stroke incidence and mortality, based on studies that adjusted traditional stroke risk factors, such as age, sex, hypertension, hypercholesterolemia, and blood glucose. Several pathophysiological mechanisms have been postulated, including endothelial dysfunction, oxidative metabolism, and platelet adhesiveness and aggregation. The role of hyperuricemia as an independent risk factor for CAD, however, remains controversial.^101^

#### 1.11.2. C-Reactive Protein

The role of inflammation in the propagation of atherosclerosis and susceptibility to adverse cardiovascular events is well established. Even though CRP is involved in the immunological process which triggers vascular remodeling and platelet deposition and is associated with increased CVD risk, there is no definitive evidence for its role as a causal factor of atherothrombosis. The Jupiter study analyzed 9,261 elderly patients of both sexes, using ultrasensitive CRP (US-CRP) levels to determine whether or not they would receive rosuvastatin; the results were similar to those found in younger individuals, namely, a reduced occurrence of cardiovascular events.^102^

Notwithstanding the publication of guidelines on the use of US-CRP for predicting CVD risk by several professional organizations, there is still a lack of consensus regarding optimal clinical use of US-CRP.^103^

#### 1.11.3. Vitamin D

Recent studies show evidence of a strong association between vitamin D deficiency and the presence of SAH, metabolic syndrome, diabetes, and atherosclerosis. It is thus considered an emerging risk factor for CVD.^104^

The mechanisms by which vitamin D exercises its role as a cardiovascular protector are still not well established. In the Third National Health and Nutrition Examination Survey (NHANES III), which involved 3,408 elderly patients, followed up for 7 years, after adjusting for cardiovascular risk, season of the year, and demographic data, verified that vitamin D levels are negatively associated with mortality risk, with this association being even stronger for cardiovascular mortality.^105^

A meta-analysis of 19 prospective studies with more than 65,000 patients demonstrated that the risk of all CVD, as well as cardiovascular death and CAD, was lower in patients with higher levels of vitamin D.^106,107^

#### 1.11.4. Genetic Factors

Aging is characterized by the complex interaction of cellular and molecular mechanisms that lead to a series of functional problems. These problems are intimately associated with one another; they include poor vasodilatation, increased arterial stiffness, and evident extracellular matrix remodeling, diffuse carotid intimal thickening, and endothelial dysfunction.

The mechanisms by which age truly contributes to cardiovascular risk continue to be the object of speculation. Although this paradigm explains vascular aging, considering classic risk factors as causal mechanisms, a recently proposed alternative view on vascular aging has emerged, which presents new mechanistic alternatives for understanding the vascular aging process. In this new paradigm, causal mechanisms of the aging process in itself, most notably genomic instability, including telomeric wear, drive the harmful changes that increasingly occur with biological aging.^108^

#### 1.11.5. Coronary Calcium Score

CCS represents an important risk marker for cardiovascular events, especially in predicting risk of AMI in subsequent years, with a score of 0 demonstrating an almost null possibility of coronary events in subsequent years. A score above 100, however, is considered an aggravating risk factor, and scores over 400 indicate a high risk of coronary events.^109^

**Table:** 

Recommendations	Grade of recommendation	Level of evidence
Coronary calcium score	IIa	C

#### 1.11.6. Investigating Subclinical Atherosclerosis

This is indicated to better stratify cardiovascular risk in elderly patients, with the aim of better identifying cases that will require more aggressive therapy. The Cardiovascular Health Study followed up elderly patients for 10 years and demonstrated that the subclinical atherosclerosis index was a better predictor of cardiovascular events than traditional risk factors in asymptomatic elderly adults. This index is composed of the ankle-brachial index (ABI), carotid artery stenosis, carotid intima-media complex thickness, altered EKG or echocardiogram, positive response to the Rose questionnaire or the intermittent claudication questionnaire.^110^ Carotid artery ultrasonography is an important resource for evaluating elderly patients. Patients with carotid blockage of 50% or more are considered at a high risk of coronary events.^111^

**Table:** 

Recommendations	Grade of recommendation	Level of evidence
Investigating subclinical atherosclerosis	I	C

##### 1.11.6.1 Ankle-brachial index

Peripheral arterial obstructive disease (PAOD) is strongly related to coronary events, and it may be assessed by the ABI, a low-cost, easily applicable exam. ABI < 0.9 is positively associated with a higher number of coronary events and with death of cardiovascular etiology. Its indication is always applicable when there are alterations in the clinical exam which suggest peripheral arterial disease, as well as excluding intermittent claudication (grade of recommendation IIa, level of evidence C). The recommendations of a recent American scientific statement highlight the strong, consistent association of advanced age with PAOD prevalence and incidence. Age > 70 is an independent risk factor for developing PAOD involving lower extremities, notwithstanding other risk factors, with a prevalence rate of > 20% in men and women in this age group. Given the strong effect of age on the prevalence of PAOD, the statement endorses the use of ABI as a class I recommendation (level of evidence C).^112^

### 1.12. Aorta and Carotid Artery Disease

#### 1.12.1. Thoracic Aortic Aneurysm

Bicuspid aortic valve (BAV) is the most frequent modality of congenital heart disease (1% to 2%), and it may occur with thoracic aortic aneurysm (TAA), with a high risk of undergoing expansion. As many as 50% of patients with BAV develop ascending aorta dilation. Factors that contribute to progression of TAA in the presence of SAH include obesity and increase in age. As these 3 conditions are frequently present together in elderly adults, TAA has been underdiagnosed in this age group. It is estimated that TAA is present in at least 3% to 4% of elderly individuals.

Patients with TAA are in primary prevention. One of the complications of TAA is acute dissection, whose frequency is 2 times higher in men than in women. Rupture, however, is responsible for 60% of deaths attributed to TAA.

Current guidelines consider a cutoff point for surgery indication for ascending TAA of 5.5 cm for patients without Marfan or BAV and 5.0 cm in the presence of one of these clinical conditions (Table 7). TAA with diameters ≥ 4 cm require annual measurement, preferably by angiotomography (gold standard, but subject to radiation) or magnetic angioresonance. In non-genetic cases of TAA of ≥ 5 cm, measurements should be performed biannually. EKG tends to underestimate aorta caliber.^113-116^

**Table 7:** Threshold diameters for indicating aortic aneurysm surgery, according to current guidelines

Aorta	Marfan/BAV	Non-marfan
Ascending	5.0 cm	5.5 cm
Descending	6.0 cm	6.5 cm

BAV: bicuspid aortic valve.

Elective TAA surgery mortality in highly specialized centers is 2.9%. The risk of stroke or paraplegia is much higher in descending aorta. In this case, the option of endovascular intervention, with stent collocation, presents lower risk of paraplegia.

#### 1.12.2. Abdominal Aortic Aneurysm

Abdominal aortic aneurysms (AAA) tend to affect elderly individuals (≥ age 65) and are atherosclerotic in nature; in this manner, AAA places patients in secondary prevention. Tobacco use is the main etiological factor of AAA, which is 3 to 5 times more common in smokers than in non-smokers. AAA is also common in patients with peripheral arterial disease (PAD).^115^

AAA is found in 1.3% of men between the ages of 45 and 54, and in 12.5% of those between the ages of 75 and 84. In women, the maximum prevalence was 5.2% in the elderly age group, being found in 0% of young women. The fact that men smoke more than women likely contributes to this pronounced difference between age groups by sex. This notwithstanding, evolution and prognosis of AAA are worse in women.^113-115^

Initial discriminatory evaluation by ultrasonography is recommended, especially in male patients who have been smokers, starting at age 65. In the event that the result is normal, there is no need for periodic reevaluation.^113-115^

AAA with diameters of ≥ 4 cm require annual measurement, which may only be performed by abdominal ultrasonography, which, in this area, has excellent sensitivity and specificity. If it is ≥ 5 cm, screening should be performed biannually. The cutoff point for indicating intervention is 5.5 cm. Open surgery poses a higher risk, but it lasts longer and should preferably be indicated in younger individuals with longer life expectancy. Endovascular intervention has evolved considerably and should preferably be indicated in older patients or patients considered high risk for surgery.^113-116^

##### 1.12.2.1. Carotid Arteries

There is no solid evidence regarding the eventual advantage of interventional treatment in intensive clinical control of cardiovascular risk factors, especially if we consider the use of full dosages of latest generation of statins, although many services opt for aggressive treatment, based only on registers and specialist opinion.^117,118^

Routine carotid ultrasonography is only indicated for patients who have suffered stroke/transient ischemic attack (TIA), or when physical examination identifies decreased, absent, or asymmetric pulse or carotid murmur.

#### 1.12.3. The Original and 10-Year CREST Studies

The original Carotid Revascularization Endarterectomy vs. Stenting Trial (CREST) study (n = 2,502) aimed to observe the medium- and long-term reduction in risk of ischemic stroke associated with carotid endarterectomy (CEA) and angioplasty with carotid-artery stenting (CAS) in patients with significant carotid atherosclerotic disease. The proportion of cerebrovascular asymptomatic patients and of those who had suffered stroke/TIA was very similar. The main study objective was to evaluate the risk of death, AMI, or stroke during the first 30 days after the procedure and of ipsilateral stroke during the following 4 years. They did not, however, find an optimized clinical treatment group. The risk of minor stroke was higher in the CAS group during the first 30 days, whereas the risk of AMI was higher in the CEA group. At the end of the 4-year period, the risk of stroke was low and similar in both groups analyzed (2.0% and 2.4%; p = 0.85). The main conclusion was that both CEA and CAS may be alternatively indicated as interventional carotid treatments. Additional findings suggest that CEA seems to be more beneficial in elderly patients, while CAS would be more useful in subpopulations under age 65.^117^

The main lesson of the 10-year CREST study was that, once the initial critical phase was over, patients who underwent interventional treatment tended to evolve very well long-term. The 10-year risk of stroke was 6.9% in the CAS group and 5.6% in the CEA group, with no significant statistical difference (p = 0.96). The primary composite endpoint (death, AMI, and stroke) occurred in 11.8% of participants in the CAS group and in 9.9% of those in the CEA group, with no statistical difference (p = 0.51). Nevertheless, the primary composite endpoint death/stroke over 10 years was worse in the CAS group (11.0% vs. 7.9%; hazard ratio [HR]: 1.37%; p = 0.04).^118^

The Asymptomatic Carotid Trial (ACT) 1 study (n = 1,453) included patients with significant asymptomatic carotid disease, randomized into interventional treatment by CEA (n = 364; 25%) or CAS (n = 1,089; 75%). Elderly patients > age and those who had suffered stroke/TIA during the past 180 days were excluded. The carotid anatomical pattern was required to be viable for both procedures, with a minimum degree of stenosis of 70% diagnosed by either ultrasonography or angiography.^119,120^

The main objective was to demonstrate the noninferiority of CAS to CEA in relation to a composite endpoint, represented by death, AMI, and stroke during the first 30 days and ipsilateral stroke within 1 year. The results during 30 days showed that the incidence of this endpoint was only 2.95%. There were more cases of stroke and stroke or death in the CAS group and more cases of AMI in the CEA subgroup. The risk of major stroke was low (0.4%) and mortality was 0.2%. Medium- and long-term survival free of stroke was excellent in both groups, 97.5% over 1 year and 93.9% over 5 years. In 5 years, 97.5% of participants did not require carotid reintervention, and total mortality was 11.8%.^119^

#### 1.12.4. Precautions and Recommendations

The main problem with interventional carotid treatment lies in the risk of death, AMI, or stroke inherent in the procedures per se, which extends to 30 days after intervention. Once this phase has passed, the annual risk of stroke or need for reintervention is considered low.

Intervention by CEA or CAS in patients with asymptomatic carotid disease dos not have a solid base for recommendation in comparison with optimized clinical treatment, and it should preferably be avoided at this moment until studies currently underway help to definitively answer this important question (CREST 2 and ACST-2).^121-123^

More than 90% of carotid interventions in the USA currently involve asymptomatic cerebrovascular patients. In Germany and Italy, these indexes are 60%; in Australia and Canada, 15%; and in Denmark, 0%.

The annual risk of stroke in asymptomatic patients with significant carotid disease receiving only clinical treatment has reached values as low as 0.5%, or be it, the same index documented in the ACT-1 and the 5 and 10 year CREST studies.^122,123^

Contrary to what has been admitted by some guidelines, it is here suggested that interventional carotid treatment be reserved for symptomatic patients (stroke/TIA over the past < 6 months), and that it be indicated for asymptomatic patients only when the degree of stenosis is between 70% and 99% in spite of optimized clinical treatment, and when there is proof that a large cerebral area is at risk or plaque-related microembolism, obtained by imaging exams and cerebral blood flow evaluation.^121-123^

### 1.13. Evaluation of Surgical Risk In Elderly Patients

The elderly population is currently growing more than any other. For this reason, a significant increase has been observed in the number of surgical procedures in this age group. The number of surgical procedures in people over age 65 is estimated to be 4 times higher than in the younger population.^124^ The prevalence of symptomatic and asymptomatic CVD increases progressively with age, as shown in the results of many studies which suggest that age ≥ 80 is an independent predictor of perioperatory complications and death in patients who undergo non-cardiac or cardiac surgery.^125^ Few studies, however, include elderly individuals over age 70 and the results are, generally, extrapolated from younger to older populations, ignoring the latter's particularities.^126^

Clinical evaluation in the elderly population should consider biological processes underlying so-called normative aging, such as physiological decrease in multiple organic functions which may cause inadequate responses to anesthetics, analgesics, and other substances administered and also lead to the appearance of cardiovascular complications, hemorrhagic or neuropsychiatric accidents, et al. It is mandatory to evaluate associated comorbidities and their repercussions on nutrition, overall functionality, independence, and healthy life expectancy, as well as all medication in use, in order both to prevent possible complications and to choose the most adequate procedure for each case.^127^

As a general rule, the establishment of a patient's surgical risk should be individualized and the bioethical principle of patient autonomy should be respected in all patient decisions or, in the event of impossibility, those of the patient's legal representative, following adequate clarification regarding the risks inherent in the disease and the surgical procedure, during the intraoperative and immediate and late postoperative periods, and the quality of life expected to result from the treatment. It is necessary to document the patient's and/or legal representative's decision in the medical records.^128^

With these considerations, surgical risk should be established based on a "tripod" comprising: (1) nature and character of the surgery; (2) functional capacity; (3) patient risk profile.

The new guidelines have established that elective and minor surgeries where the possibility of heart attack or major adverse cardiovascular events is ≤ 1% are low risk; when the possibility is ≥ 1% they are considered high risk. More recent publications have incorporated intermediate or high risk.^129^ Patients indicated for urgent surgery should have their risks established when possible, using information provided by the family or the patients themselves, and then be referred to the surgical center. In the event of elective surgeries where the patient's hemodynamic conditions are not stable, they must be treated before establishing status and choosing the most opportune moment to perform the operation.

Patient functional capacity is a valuable indicator of risk of complications during the course of surgery and the postoperative period. The ability to ascend 2 stories by stairs or by ramp or to walk at a velocity of approximately 4 mph on a level surface corresponds to a metabolic equivalent (MET) ≥ 4, which indicates a good cardiovascular reserve and regular physical capacity; MET ≥ 10 is considered very good.

The last step in this strategy is to establish the patient's risk profile based on his or her clinical history, symptoms, signs, and laboratory data. In the presence of unstable coronary syndromes, decompensated HF, symptomatic valve disease, severe arrhythmias, or pulmonary embolisms which may compromise the course of the perioperative period, non-invasive exams are indicated in order to improve comprehension. When non-invasive exams are suggestive of coronary insufficiency, it is necessary to indicate scintigraphy stress testing, eventual coronary angiography, and even myocardial revascularization, provided that performing this may substantially change patient management or survival, taking the severity of the underlying disease into account.^130^

### 1.14. Vaccination in Elderly Patients

#### 1.14.1. Brazilian Immunization Society (SBIm) Recommendations - 2015/2016^131^

**Influenza** [indicated for all elderly individuals] - Influenza is a highly infectious acute respiratory infection, caused by *Myxovirus influenzae*, a virus that is not specific to humans (The virus infects different domestic and wild vertebrates which may, in turn, infect humans). There are 3 known types, A, B, and C, and there is no crossed immunity between them. Type A is the most virulent. It causes the largest epidemics, and is subdivided into subtypes in accordance with the characteristics of its superficial molecules (designated by the abbreviations HA and NA). There are currently 2 subtypes of influenza A in circulation among humans: H1N1 and H3N2.

The mortality associated with this virus may be elevated in more elderly and very young individuals, as well as in those with respiratory, cardiovascular, or renal pathologies, or diabetes, for example. The severity of the illness may be due to the virus itself or, more frequently, overlapping bacterial infections that follow influenza. There are 2 types of influenza vaccine, trivalent (3V) and quadrivalent (4V). The 3V protects against the H1N1 and H3N2 strains (both influenza A) and against a 1 type of the influenza B virus. The 4V protects against the forenamed strains and, additionally, against a second influenza B virus. Provided that it is available, the 4V influenza vaccine is preferable to the 3V, as it provides greater protection against circulating strains. If it is not possible to use the 4V vaccine, the 3V vaccine should be used. The vaccine offered by the public system is the 3V. Contraindications include known systemic hypersensitivity to any medication or substance, including neomycin, formaldehyde, triton-X-100 (octoxinol 9), eggs, or chicken protein, either following the administration of this vaccine or a vaccine containing the same composition. People with acute febrile diseases should not, normally, be vaccinated until these symptoms have disappeared.

**Pneumococcal vaccine** [indicated for all elderly individuals] - This vaccine protects against invasive infections (sepsis, meningitis, pneumonia, and bacteremia) and acute otitis media (AOM), caused by some serotypes of *Streptococcus pneumoniae*. It starts with a dose of VPC13, followed by a dose of VPP23 6 to 12 months later, and a second dose of VPP23, 5 years after the first. For those who have already received VPP23, an interval of 1 year is recommended for the application of VPC13. A second dose of VPP23 should be given 5 years after the first, maintaining an interval of 6 to 12 months after the dose of VPC13. For those who have already received 2 doses of VPP23, a dose of VPC13 is recommended at a minimal interval of 1 year after the latest dose of VPP23. If the second dose of VPP23 was applied before age 65, a third dose is recommended after this age, with a minimum interval of 5 years after the latest dose. This vaccine is available through the public system for risk groups (COPD, diabetes, etc.)

**Diphtheria, tetanus, and acellular pertussis (DTaP)/diphtheria and tetanus (DT)** [indicated for all elderly patients] - This vaccine protects against diphtheria, tetanus, and acellular pertussis (DTaP) or diphtheria and tetanus (DT). A DTaP booster is necessary, regardless of previous DT or tetanus interval. For elderly patients who intend to travel to countries where polio is endemic, the combined DTaP inactivated poliovirus vaccine (DTaP-IPV) is recommended. The combined DTaP-IPV vaccine may substitute the DTaP. When the basic vaccination schedule for tetanus is complete, a DTaP booster is recommended every 10 years. When the basic vaccination scheme for tetanus is incomplete, a DTaP dose is recommended at any moment, completing basic vaccination with 1 or 2 doses of adult DT vaccine, in a manner that totals 3 doses of tetanus vaccine. This vaccine is recommended, even in individuals who have already had pertussis, given that protection provided by the infection is not permanent. It is possible to consider anticipating a DTaP booster, containing the pertussis component, to 5 years after the latest dose in elderly individuals who are in contact with breastfeeding infants. The DT is available through the public system.

**Herpes zoster** [indicated for all elderly patients] - This vaccine is recommended even in patients who have already had herpes zoster. In these cases, a minimum interval of 1 year is necessary between the acute phase and the vaccine application. In cases of patients with a history of ophthalmic herpes zoster, there are still not enough data to indicate or contraindicate the vaccine. Regarding use in immunocompromised patients, the vaccine should not be used in individuals with primary or acquired immunodeficiency states or those undergoing drug therapy at doses considered immunosuppressive. This vaccine is not available through the public system.

#### 1.14.2. Other Vaccines (Non-Routine)

**Hepatitis A, B, or A+B** - Hepatitis A: 2 doses, in 0 and 6 month schedule. Hepatitis B: 3 doses, 0, 1, and 6 month schedule. Hepatitis A and B: 3 doses, 0, 1, and 6 month schedule. For hepatitis A, in the over 60 population, susceptible individuals are not commonly found. Vaccination is, thus, not a priority in this group. Serology may be requested in order to determine whether or not to vaccinate. In patients who have contact with hepatitis A or during an outbreak of the disease, vaccination should be considered. Regarding hepatitis A, B, and A+B, the combined hepatitis A and B vaccine is an option, and it may substitute isolated vaccination for hepatitis A and B.

**Yellow fever** - The vaccine is necessary in residents of risk areas and in those who intend to travel to these areas, at least 10 days before travel. If the risk persists, 10 years later, a second dose is necessary. This vaccine is contraindicated in immunocompromised individuals; however, when the risks of acquiring the disease outweigh the potential risks associated with vaccination, the physician should evaluate its use. There are reports of a higher risk of serious adverse events in patients over 60 years of age; therefore, if it is the primary vaccination, it is necessary to assess the risk-benefit ratio.

**Measles, mumps, and rubella** - Individuals are considered protected when they have, at some point in their lives, over 1 year of age, received 2 doses of the measles, mumps, and rubella vaccine with a minimum interval of 1 month between them. The vaccine is indicated in increased risk situations, given that the majority of people in this age group are not susceptible to these diseases. In the over 60 population, individuals susceptible to measles, mumps, and rubella are not commonly found. In this group, vaccination is thus, not routine. Nonetheless, according to medical criteria (during outbreaks, before travel, et al.), it may be recommended. It is contraindicated in immunocompromised individuals.

### 1.15. Palliative Care

Palliative care (PC), which was initially focused on oncology, has been incorporated into diverse practice areas, one of which is cardiology, with discussions on PC in the area of CVD, especially involving the most elderly population. For this reason, this topic deserves to be covered in this document.

According to the WHO, PC is defined as a mode of assistance provided by a multidisciplinary team with the objective of improving patient and family member quality of life, when faced with a life-threatening disease, through prevention and relief of suffering.^132^ PC requires early identification, evaluation, and treatment of pain and other physical, social, psychological, and spiritual issues.^132,133^

PC should be individualized; it is not an approach to "terminal" patients, but rather to a life-threatening clinical condition.^133^ Its indication should be early, at the moment of diagnosis, in a manner that promotes understanding, acceptance, and progressive expansion of the means of support over time. The possibility of whether or not to implement disease-modifying treatments should be discussed in a manner that does not allow for the idea that "there's nothing to do."^133^

The principles that guide PC in accordance with the WHO consist of:^132^

1. Relieve pain and other distressing physical symptoms.
2. Affirm life and consider death as a normal life process.
3. Neither hasten nor postpone death.
4. Integrate psychological and spiritual aspect into patient care.
5. Offer a support system that makes it possible for the patient to live as actively as possible, until the moment of death.
6. Offer a support system that helps family members cope with the disease and bereavement.
7. Improve quality of life and positively influence the course of the disease.
8. Initiate care as early as possible, in conjunction with other life-prolonging measures, such as chemotherapy and radiotherapy, and include all necessary investigations to better comprehend and control existing clinical situations.

From the theoretical point of view, all patients with serious, incurable, and progressive diseases that are life-threatening should receive PC.^133^ If this reference were put into practice, the number of patients indicated for PC would be enormous, and it would not be possible to provide this type of assistance to all of them. For this reason, the National Academy of Palliative Care (Academia Nacional de Cuidados Paliativos, ANCP)^133^ recommends the adoption of the criteria used by Medicare in the United States,^134^ which establishes expected survival time as a criterion for indicating PC. Adapting the Medicare criteria, we may suggest the following conditions for indicating PC:^133,134^

1. Patient with life expectancy less than or equal to 6 months.
2. Diagnosis with an incurable and irreversible disease.
3. The patient must opt for PC, giving up life-prolonging treatments.
4. The implementation of PC should be operationally available.

Prognostic evaluation of patients receiving PC is a complex process involving physiological and social judgments. The ANCP recommends some instruments for evaluating patient functionality, as well as measuring functional and clinical decline, such as the Karnofsky Performance Status Scale and the Palliative Performance Scale. These scales and their methods of evaluation are detailed in the ANCP's Palliative Care Manual, which is available on their virtual library (http://paliativo.org.br/).^133^

In relation to CVD, they are known to be the main cause of death in Brazil, as well as in other parts of the world. They may occur at any age, but their prevalence is higher with advanced age.^133^ Among CVD, HF represents a challenge to prognostic evaluation, given that many patients die suddenly, even when they are in higher functional classes. Diverse criteria have sought to identify patients with HF at a risk of sudden death, such as left ventricular ejection fraction (LVEF), type B natriuretic peptide, end-diastolic LV diameter, presence of nonsustained ventricular tachycardia, diabetes mellitus, thromboembolic phenomena, history of previous cardiorespiratory arrest, and AIDS diagnosis.^133^ The difficulty of prognosis in patients with HF makes it challenging to discuss care preferences with patients; for this reason, these patients have been considered those with the least comprehension of their clinical condition and the least involved in the decision making process related to their care.^133^ Patients with CVD suffer severely, and they are among those who least receive home healthcare and PC; for this reason, these Guidelines agree with the idea that PC should be considered earlier during the evolutionary course of CVD and in routine cardiology practice.

## 2. Chronic Coronary Disease

### 2.1. Peculiarities of Diagnosing Chronic Coronary Artery Disease in Elderly Patients

Clinical history and detailed physical examination are essential when evaluating an elderly patient with suspected chronic CAD; however, in routine practice, this constitutes a challenge, considering the occurrence of comorbidities, atypical symptoms, and alterations in cognition and locomotion.

Ischemia is frequently present in the form of anginal equivalents, such as fatigue, dyspnea, and epigastric discomfort, with the presence of typical angina being rare.^135^ Physical examination, generally non-specific, may provide some leads, such as SAH, abnormal heart rhythms such as atrial fibrillation (AF), and peripheral arterial disease.

Resting EKG may be non-specific in 50% of cases, even in those with severe coronary disease;^136^ alterations such as pathological Q waves, T-wave inversions, left ventricular hypertrophy (LVH), His bundle branch blocks, and AF are common in elderly patients. These alterations complicate diagnosis. EKG is particularly useful during episodes of angina, when ST segment depression or pseudonormalization may be observed in up to 50% of cases.

Chest radiography should be performed when there is a suspected coexistence of congestive HF, valvulopathy, or respiratory disease.

Transthoracic echocardiography provides information which is relevant to diagnosis and management of chronic: (a) LV status - systolic and diastolic function, parietal mobility, and hypertrophy; (b) presence of valvulopathy; (c) situation of the aortic root.

The use of functional tests for ischemia (ET, stress echocardiography and myocardial perfusion scintigraphy [MPS]) or anatomical tests (coronary computed tomography angiography [CCTA] and coronary cine angiogram [CCA]) depends on pre-test estimates on the likelihood of obstructive CAD.^137^ When the probability is low (< 20%), it is not necessary to continue investigation. On the other hand, when the probability is high, (> 80%), negative results of non-invasive exams cannot exclude obstructive CAD; invasive strategies may, thus, be considered. In patients with intermediate pre-test probability, a stress test is indicated.

In elderly patients, the diagnostic sensitivity and specificity of ET have been questioned,^138^ as a result of low exercise capacity (reduced muscle mass, deconditioning, comorbidities) and the presence of alterations in baseline EKG; nevertheless, this method may be useful in clinical management, offering relevant information on symptoms, exercise capacity, chronotropic response, arrhythmias, etc.

Both stress tests and MPS may be used in association with the ET to increment sensitivity and specificity for ischemia.^139,140^ Diagnosis and prognosis of both modalities are similar and the preference for a determined method depends on the experience and/or equipment available at the investigating center. For elderly patients incapable of exercising, pharmacological stress may be used both in the stress test (dobutamine) and the MPS (vasodilatory agents).

The CCS, obtained in conjunction with CCTA, is useful for risk stratification in asymptomatic elderly patients, due to its high negative predictive value;^141^ its value, however, is limited in symptomatic patients with suspected CAD. Due to the high prevalence of coronary calcification in the elderly, CCTA has shown to be of reduced accuracy in demonstrated obstructive CAD.^142^

CCA continues to be the "gold standard" for definitive evaluation of epicardial CAD; it is generally recommended for patients whose clinical characteristics and/or non-invasive test results indicate a high likelihood of severe coronary disease, with a high risk of coronary events or death. Even though it is well tolerated, it deserves attention due to the risk of bleeding, stroke, and contrast-induced nephropathy.

### 2.2. Peculiarities of Treating Chronic Coronary Artery Disease In Elderly Patients

During the last decades, the treatment of coronary disease has been founded on general clinical measures related to the development of healthy habits, such as a balanced diet, weight control, regular practice of physical activity, vaccination schedule completion, tobacco cessation, intensive BP control, and appropriate use of antiatherosclerotic medications such as statins, antiplatelet medications, and renin-angiotensin system inhibitors, in addition to antianginal agents.^143-145^ Additionally, well selected cases are treated with myocardial revascularization procedures, through percutaneous coronary intervention or surgery. In elderly patients, these principles are largely applicable with evidence which it has been possible to extrapolate from randomized clinical trials, that have begun to include "young" elderly individuals (ages 60 to 75) in their observations, with less frequently evaluation of "truly elderly" individuals (ages 75 to 85) are scarce evaluation of "very elderly" individuals (over age 85).^143-145^

Regarding diet, the Lyon, Dietary Approaches to Stop Hypertension (DASH), and, more recently, *Prevención con Dieta Mediterránea* (PREDIMED) studies have validated the concept of a healthy diet; the PREDIMED included patients up to age 80. Weight control represents a particular consideration in the elderly owing to the apparent existence of a paradox between BMI and age.^146^ In a more conclusive analysis of the topic of CAD, the reduction of obesity is associated with better results.

Regular practice of activities which are appropriate for the elderly individual's physical conditions bring innumerable psychological benefits that impact improvements in general healthcare and which justify their implementation.

Inflammation caused by infections plays a recognized role on the emergence of coronary disease complications, and influenza and pneumococcal vaccination is a recommendable measure in elderly coronary disease patients.^147^

Analysis of the Coronary Artery Study (CASS) registry has been definitive in demonstrating the benefits of tobacco cessation in elderly coronary disease patients.^148^

A systolic blood pressure (SBP) control goal of < 140 mmHg has been established for the elderly population. A recent study, the Systolic Blood Pressure Intervention Trial (SPRINT), recommends that this goal be even more intensive, even in elderly coronary disease patients (< 130 mmHg, if tolerated), without verifying the J curve or undesired events in relation to reduced diastolic BP. Special caution needs to be taken in this population when comorbidities are present.^149^

Antiatherosclerotic medications such as statins have confirmed demonstration, in clinical trials, up to age 79. If tolerated, they should be used to stimulate an LDL-c goal of < 70 mg/dL. Acetylsalicylic acid (ASA) is recommended, as well as the use of angiotensin converting enzyme inhibitors (ACEI) or angiotensin receptor blockers (ARB), even in the absence of SAH or HF, notwithstanding the fact that both of these conditions are frequently associated with CAD in the elderly.

Anti-ischemic medications, such as beta-blockers (and calcium channel blockers, when beta-blockers are not possible, or in association with them) for control and nitrates for crises, as well as new anti-ischemic medications, such as trimetazidine, should be used with due caution regarding progressive doses, due to the higher incidence of side effects. Ivabradine may be considered for HR control when it is not possible to use beta-blockers.^150^

In relation to revascularizing elderly patients without frailty by either percutaneous or surgical intervention, this should be considered with the aim of controlling refractory symptoms or in cases with severe ischemic burden. With respect to deciding which procedure should be performed, whether percutaneous intervention or surgery, this depends wholly on the feasibility of using the techniques, it being necessary to consider that age adds a considerable weight to risk of both procedures and that scores that include associated comorbidities tend to affect the surgical procedure even more.^151^

In conclusion, in addition to the previously mentioned facts, therapeutic recommendations must consider many other relevant factors such as biological aspects of frailty, psychological competence, economic and social support, among others. This makes this choice an optimal example of personalized therapy centered on the elderly individual who is affected by CAD.

### 2.3. General Recommendations - Chronic Coronary Artery Disease in Elderly Patients

**Table:** 

Diagnostic evaluation of chronic coronary disease in elderly patients				
Method	Positive aspects	Possible limitations	Grade of recommendation	Level of evidence
EKG	Easily obtained. Detection of inactive zones and conduction disorders	Low accuracy	I	B
Ergometric test	Availability. Moderate accuracy in detecting ischemia	Locomotive difficulties. Resting EKG alterations	I	B
Stress echocardiography (exercise, dobutamine, or dipyridamole)	Detection and evaluation of the extent of ischemia. Evaluation of LV function	Echocardiography window. Cost	I	B
Scintigraphy	Detection and evaluation of the extent of ischemia. Does not depend on preexisting electrocardiographic alterations. Evaluation of LV function	Lower availability. Cost	I	B
Coronary computed tomography angiography	Detection of obstructions	Calcification in the elderly patient decreases diagnostic accuracy	IIa	B
Coronary magnetic resonance angiography	Detection of obstructions	Lower accuracy. Difficult to obtain	IIb	C
Cardiac magnetic resonance	LV function. Areas of fibrosis	Difficult to obtain	IIb	C

EKG: electrocardiogram; LV: left ventricle.

**Table:** 

Treatment of chronic coronary disease in elderly patients	
General measures	● Balanced diet ● Weight control ● Regular practice of physical activity ● Vaccination schedule completion ● Tobacco cessation ● Intensive blood pressure control
Antiatherosclerotic medications	● Statins ● Antiplatelet ● Renin-angiotensin system inhibitors (ACEI/ARB)
Antianginal medications	● Beta-blockers ● Calcium channel blockers ● Nitrates ● Trimetazidine
Myocardial revascularization	● Percutaneous coronary intervention ● Myocardial revascularization surgery

ACEI: angiotensin converting enzyme inhibitors; ARB: angiotensin receptor blockers.

**Table:** 

Recommendations for general measures and antiatherosclerotic use		
Procedure/medication	Grade of recommendation	Level of evidence
Balanced diet	I	A
Weight control	I	B
Physical activity	I	B
Vaccination against influenza	I	B
Tobacco cessation	I	A
BP control < 140 mmHg	I	A
BP control < 120 mmHg	IIa	B
Statins	I	A
Antiplatelets	I	A
ACEI/ARB	I	A

ACEI: angiotensin converting enzyme inhibitors; ARB: angiotensin receptor blockers. BP: blood pressure.

**Table:** 

Recommendations for antianginal medications		
Medication	Grade of recommendation	Level of evidence
Beta-blockers	I	A
Calcium channel blockers	IIa	B
Nitrates for anginal crises	I	A
Nitrates for chronic use	IIb	B
Trimetazidine	IIa	B
Ivabradine	IIa	B

**Table:** 

Indication for revascularization in elderly patients refractory to clinical treatment		
PCI – Patients with angina	Grade of recommendation	Level of evidence
PCI feasible and easily applied	I	C
Low SYNTAX score	I	B
High SYNTAX score	IIb	B
**Surgery – Patients com angina**	**Grade of recommendation**	**Level of evidence**
Multivascular, with low surgical risk	I	B
Low SYNTAX score and moderate to high surgical risk	IIb	B

PCI: percutaneous intervention; SYNTAX: Synergy Between Percutaneous Coronary Intervention with Taxus and Cardiac Surgery.

**Table:** 

Indication for revascularization in asymptomatic elderly patients		
Severe ischemic load	Grade of recommendation	Level of evidence
Percutaneous intervention	IIa	C
Surgery	IIa	C

## 3. Acute Coronary Disease

### 3.1. Diagnostic Peculiarities

Elderly patients have a higher incidence of acute coronary syndrome (ACS), and their prognosis is worse in comparison with younger patients. The causes of this unfavorable evolution include: (a) delayed arrival at the hospital; (b) diagnostic difficulties; (c) lower likelihood of receiving interventional treatment; (d) less use of beta-blockers; (e) previous HF; and (f) comorbidities.^152^ As age increases, the effects of risk factors such as hypertension, diabetes, and tobacco use decreases, and the importance of associated comorbidities, such as stroke and renal and cardiac insufficiency increases.^153,154^ Atypical presentation is more common in this age group; chest pain is present in 40% of patients ≥ age 80, compared to 80% in those ≤ age 65. In elderly heart attack patients, 8.4% do not present precordial pain (43.3% in patients ≥ 75 years old, compared to 29.4% in those ≤ 65 years old). More common symptoms include: dyspnea (29.4%), sweating (26.2%), nausea and vomiting (24.3%), and syncope and pre-syncope (19.1%), which are denominated ischemic equivalents.

Although physical examination may be normal, the presence or absence of signs of peripheral hypoperfusion, vital signs, presence or absence of arterial pulses, jugular vein distention, cardiac auscultation (blowing, friction, third heart sound), and pulmonary auscultation with signs of congestion are important data to evaluate. Initial EKG is less solicited and more delayed, in elderly patients: 40% of patients ≥ age 85, compared to 25% of those ≤ age 65, do not have diagnostic EKG. The presence of non-specific EKG alterations and blocks is more frequent in elderly patients, increasing diagnostic difficulties in this age group, especially in the presence of left bundle branch block.^102,155^ Elevated myocardial necrosis markers unrelated to ACS are common in other situations, such as increased plasma N-terminal brain natriuretic propeptide (NT-pro-BNP), diabetes, renal insufficiency, anemia, dehydration, metabolic and hydroelectrolytic disorders, infections, and echocardiography abnormalities in chronic heart diseases.^156-159^

Risk scores, such as the Thrombolysis in Myocardial Infarction (TIMI) Risk^160^ and the Global Registry of Acute Coronary Events (GRACE),^161^ are important for risk stratification of elderly ACS patients, ensuring better strategy in diagnostic and therapeutic approach, increasing the use of antithrombotic and anticoagulant medications and myocardial revascularization, with a consequent decrease in risk of death, heart attack, and recurring ischemia.^162,163^ Being over age 70 confers a moderate (ages 70 to 75) to high (> age 75) risk of coronary disease.

Frailty is an important independent predictor of mortality, longer hospital stays, increased risk of bleeding and morbidity in the elderly population with ACS.^164,165^ Functional decline in elderly patients is a predictor of poor evolution.^166^ The Gold Standards Framework (GSF) score, which associates end-stage disease criteria, has shown to be an independent predictor of non-cardiovascular events in ACS, while the GRACE score has demonstrated that it is an excellent predictor of cardiovascular events in elderly patients.^167^ Chest radiography, resting transthoracic echocardiography, myocardial scintigraphy, coronary angiotomography, cardiac magnetic resonance, and CCA follow the same indications as in younger patients for diagnosis of ACS in this age group.^156,157^

### 3.2. Peculiarities of Treatment

Even though the elderly population is the one that most benefits from more aggressive strategies, they have a higher risk of bleeding, with a 2-fold risk of mortality compared to younger patients (< 75 years old). Higher intra-hospital mortality and higher bleeding rates with thrombolytic therapy are part of this scenario. Approach to ACS in elderly patients should be individualized, based on risk of complications, estimated life expectancy, comorbidities, quality of life, and the patient's wishes and preferences.^153,154,156,157,168-170^ Elderly patients (> age 75) with acute coronary syndrome with ST-segment elevation (ACS-STE) and without ST-segment elevation (ACS-NSTE) should follow the same diagnostic and therapeutic approach as in younger patients, based on guidelines and consensuses, it being necessary to evaluate particularities of pharmacokinetics, sensitivity, and collateral effects and collateral effects of drugs, always taking weight and creatinine clearance into account.^102,153,154,156,157,168-170^

During the past 15 years, there has been a significant increase in the rates of pharmacological therapy use based on evidence for ACS patients in all age groups. However, in cases of ACS-STE, elderly patients have a lower chance of receiving primary angioplasty or thrombolysis, as well as the prescription of ASA, clopidogrel, beta-blockers, statins, or ACEI.^171^ The Study of Global Ageing and Adult Health (SAGE) compared the effects of intensive (atorvastatin 80 mg) versus moderate statin therapy (pravastatin 40 mg) on reducing myocardial ischemia in elderly patients between the ages of 65 and 85. Both statin regimens were equally effective in reducing the frequency and duration of ischemia; intensive therapy with atorvastatin, however, was demonstrated to be more effective in the reduction of lipids and all-cause mortality, in comparison with pravastatin.^170,172^ However, due to the prevalence of collateral effects and intolerance to this medication in this age range, lower doses of statins are suggested for ACS patients, until LDL-c < 70 mg/dL has been reached, maintaining the tolerated dose.

After age 85, studies suggest that there are benefits associated with reperfusion strategies for ACS-STE. The choice between fibrinolytic drugs and angioplasty is determined by the presence or absence of cardiogenic shock, presentation time, and comorbidities, which often tend toward angioplasty in elderly patients. The safety and efficacy of reperfusion, especially fibrinolytic therapy, in very elderly patients (≥ 85 years old) are questions which require deeper investigation.^173^ The After Eighty study investigators evaluated 457 patients over the age of 80 with ACS-NSTE (AMI and unstable angina) who were randomized to an invasive or a conservative strategy, suggesting that invasive therapy is superior, with a higher incidence of death, myocardial infarction, and stroke in the conservative therapy group. The same results were obtained in the subgroup of elderly patients over age 90.^174^

### 3.3. General Recommendations - Acute Coronary Syndrome in Elderly Patients

With elderly ACS patients, cardiologists face the following 3 challenges:

**Table:** 

1^st^ Challenge: summary of diagnostic challenges in elderly patients
Atypical presentation: less typical pain and more anginal equivalents (dyspnea, syncope, stroke, HF, etc.)
Greater severity: present with more HF and cardiogenic shock
Higher prevalence of morbimortality: reinfarction, stroke, more severe hemorrhage, and death
Lower effects of risk factors and greater importance of comorbidities
Non-specific EKG in 43% of elderly patients > 85 years old
Myocardial infarction (ACS-STE) should be strongly suspected in women, diabetes patients, and elderly patients with atypical symptoms
Due to frequent atypical presentation, elderly patients (> 75 years old) should be investigated for ACS-NSTE with a lower level of suspicion

**Table:** 

2^nd^ Challenge: summary of challenges regarding approach individualization
Heterogeneous population
Moderate to high risk in the most utilized risk stratification scores (TIMI, GRACE)
Treatment should consider overall health, comorbidities, cognitive status, life expectancy, frailty, patient's wishes and preferences
It is necessary to pay attention to pharmacokinetic alterations and sensitivity to hypotensive drugs

**Table:** 

3^rd^ Challenge: summary of treatment challenges
Treat elderly patients (≥ 75 years old) with medical therapy, early invasive strategy, and revascularization, as indicated, in accordance with guidelines
It is necessary to pay attention to adjustments in doses of antithrombotic drugs in elderly patients and patients with renal insufficiency
Antithrombotic treatment should be adapted in accordance with weight and creatinine clearance
Intensive medication strategies and revascularization intervention strategies should always be considered, observing the adverse effects of these therapies
Adjustments in doses of beta-blockers, ACEI, ARB, and statins should be considered, with the aim of decreasing or avoiding collateral effects
Consider invasive strategies and, if appropriate, revascularization, following careful evaluation of potential risks and benefits, estimated life expectancy, comorbidities, quality of life, frailty, and patient preferences
It is reasonable to choose myocardial revascularization surgery over angioplasty in more elderly patients, especially those with diabetes or multiple vessel disease, due to increased survival and reduction of cardiovascular events

ACEI: angiotensin converting enzyme inhibitors; ACS-NSTE: acute coronary syndrome without ST-segment elevation; ACS-STE: acute coronary syndrome with ST-segment elevation; ARB: angiotensin receptor blockers; GRACE: Global Registry of Acute Coronary Events; HF: heart failure; TIMI: Thrombolysis in Myocardial Infarction.

## 4. Heart Failure

### 4.1. Diagnostic Peculiarities of Heart Failure in Elderly Patients

**Figure 1:**
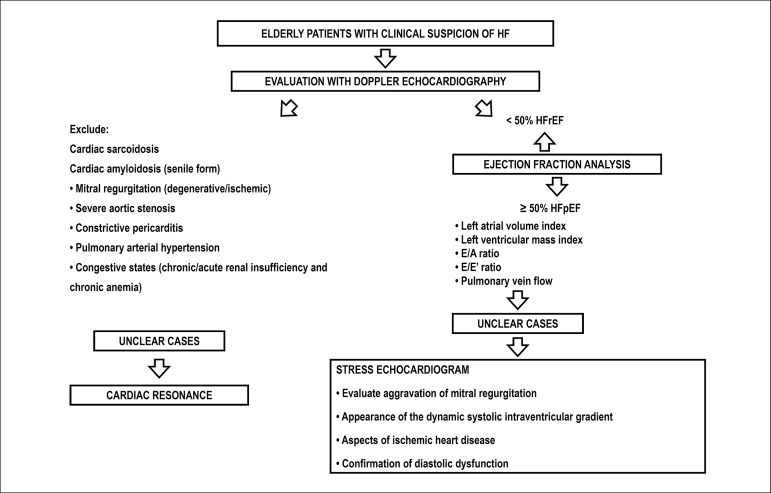
Diagnostic flowchart. HF: heart failure; HFpEF: heart failure with preserved ejection fraction; HFrEF: heart failure with reduced ejection fraction.

Elderly patients may have atypical presentations of HF due to cognitive alterations, sedentarism, functional limitations, and the presence of comorbidities. These factors contribute to late diagnosis, thus making complementary exams important (Figure 1).^175^ The use of biomarkers, such as outpatient values of brain natriuretic peptide (BNP) below 35 ng/mL, excludes the presence of HF in symptomatic individuals. In individuals with acute dyspnea in the emergency room, however, BNP values over 250 ng/mL or pro-BNP over 1,800 ng/mL indicate HF as the cause of the symptoms. Elderly patients have higher natriuretic peptide levels, as well as comorbidities which may increase these values, such as renal insufficiency.^176^ Normal EKG results may be useful in making the hypothesis of HF less likely, while findings of AF, complete left bundle branch block, inactive areas, and LVH, increase the probability of this disease.^176,177^ Alterations in cardiac geometry and structure occur with aging, including decreases from the base to the apex, right deviation, aortic annulus dilation, and increased interventricular septum thickness, which leads to so-called Sigmoid septum and may cause outflow obstruction.^176^ Even though patients with HF with reduced ejection fraction (HFrEF) (LVEF < 40%) and HF with preserved ejection fraction (HFpEF) (LVEF > 50%) are well defined, there is some uncertainty in elderly patients with moderate HF (LVEF 41% to 49%). A recent study demonstrated that this intermediate profile is a distinct entity and it should be categorized as HFrEF due to the elevated prevalence of coronary disease and to the similar benefits of using the standard of treatment indicated for this biomarker.^178^ Echocardiography study allows for evaluation of indexed left atrial (LA) volume, the presence of LV hypertrophy, analysis of filling pressures (E/A ratio, E/E' ratio, and pulmonary flow), diastolic function, inferior vena cava variation, pulmonary BP evaluation, degree of mitral regurgitation, and the presence or absence of aortic stenosis (AS) (especially the low-flow, low-gradient phenotype with normal ejection fraction). It also allows for investigation of etiology, where senile amyloidosis is currently a growing condition in individuals over the age of 70.^176,177,179^ In clinical practice, evaluation of functional state using ergospirometry aids prognostic evaluation and cardiac rehabilitation planning. The presence of fibrosis, cardiac hypertrophy, cardiac chamber dilation, intracardiac thrombus, pericardial thickening, in addition to the study of right ventricle (RV) function, may be evaluated by cardiac resonance. This has become an integral part of the evaluation of myocardial disease patients, as it identifies the cause (inflammation [myocarditis], amyloidosis, sarcoidosis, Chagas disease), cardiomyopathies, and ischemic disease.^176^ Myocardial scintigraphy is a useful method in individuals with suspected ischemic heart disease with systolic dysfunction; it is requested to investigate ischemia and/or myocardial viability. Technetium pyrophosphate bone scintigraphy may be useful in diagnosing transthyretin cardiac amyloidosis in elderly hypertrophy and HF patients.^176^

**Table:** 

Intervention	Recommendation	Grade of recommendation	Level of evidence
Oxygen	In patients with arterial saturation below 90%, respiratory failure, or a high risk of hypoxemia, it is necessary to maintain during the first 6 h or until hemodynamic stabilization is reached	I	C
Nitrates	In sublingual form, it is recommended for patients with ischemic type chest pain. It may be used in intravenous form in elderly patients with persistent pain and conditions associated with hypertension and heart failure. It should be avoided in cases of hypotension, right ventricular infarction, and severe aortic stenosis	I	C
Morphine	This should be reserved for patients with unacceptable pain levels. The initial dose is 2 to 4 mg, with 2 to 8 mg increments repeated in 5 to 15 minute intervals	I	C
Beta-blockers	Great benefits in comparison with younger groups, regarding prevention of ACS and death. Intravenous administration should only be used in specific cases	I	B
ACEI	Benefits especially in CHF or LV dysfunction	I	A
Statins	Dyslipidemia treatment in elderly patients up to age 75 should follow the same orientations as in non-elderly patients	I	A
	After age 75, doses of lipid-lowering agents should be individualized according to the presence of comorbidities, life expectancy, and polypharmacy	I	B
ASA	Indicated for all elderly patients, if there are no contraindications. Benefits are greater in elderly patients	I	A
Clopidogrel	Indicated for elderly ACS patients with high risks, especially those who will undergo angioplasty. Loading doses are not recommended in elderly patients who are eligible for thrombolytic therapy	I	A
Ticagrelor	Better evolution than clopidogrel, comparing groups over and under age 75, with no differences in bleeding in either of the 2 groups	I	B
Prasugrel	Contraindicated in patients ≥ 75 years old, weight < 60 kg, and stroke/TIA history	III	A
Antithrombins	Should be administered with caution in ACS patients. Enoxaparin may be administered at reduced doses in patients > 75 years old (0.75 mg/kg, SC, 12/12h)	I	A
Glycoprotein inhibitor IIb/IIIa	Indicated in the most elderly subgroups at the moment of intervention, excluding renal insufficiency:	I	A
	ACS-NSTE – Early intervention strategies, when thienopyridine is not administered	IIa	C
Thrombolysis	When indicated, evaluate with attention to contraindications, as they are more frequent in elderly patients. In the event of tenecteplase use in elderly patients > age 75, administer a half-dose	I	A
Primary angioplasty	Better risk-benefit compared to thrombolytic drugs	I	A
Early catheterization	Improved short- and long-term evolution. Evidence from randomized, controlled studies are limited in elderly patients and should take risk of bleeding into account. Data are lacking in the ≥ age 80 subgroup	IIa	B
	ACS-STE – Elderly patients should be considered for early invasive strategies, with the possible option of revascularization	I	A
Cardiac rehabilitation	The same benefits as in younger groups regarding death prevention	I	B

ACEI: angiotensin converting enzyme inhibitors; ACS: acute coronary syndrome; ACS-NSTE: acute coronary syndrome without ST-segment elevation; ACS-STE: acute coronary syndrome with ST-segment elevation; ASA: acetylsalicylic acid; CHF: congestive heart failure; LV: left ventricle; TIA: transient ischemic accident.

### 4.2. Peculiarities of Heart Failure Treatment in Elderly Patients

HF is prevalent among the elderly, affecting up to 20% of patients > 75 years old.^1^ It is characterized by the presentation of systolic dysfunction (HFrEF) or diastolic dysfunction (HFpEF) and high mortality (2-fold risk of all-cause mortality adjusting for age and sex and 4-fold risk of cardiovascular death).^180,181^ Over the past decades, HFpEF has become the main clinical phenotype.^2^

Polypharmacy is extremely common in this context, with a strong impact on drug interactions, higher rates of adverse effects and poor adherence; however, multidisciplinary and adherence programs have been shown to be useful in this group of patients.^182^ Exercise training, in comparison with habitual care, in elderly HFrEF patients in New York Heart Association (NYHA) classes II and III, was shown to be safe, without an increase in mortality and hospitalization and with improvements in the walking test.^183^

The objectives of pharmacological treatment of HF are: reducing mortality and hospitalization; improving functional capacity and quality of life; and including the use of ACEI, ARB, beta-blockers, and aldosterone antagonists. Elderly individuals have frequently been excluded or under-represented in studies performed on HF patients.^184^

Several influential clinical trials have demonstrated the efficacy of ACEI in younger patients (average age of 60/66); however, subgroup analysis of the Heart Outcomes Prevention Evaluation (HOPE) study demonstrated a higher risk reduction in patients > 65 years old, in comparison with the younger group.^184^

ARB have been little evaluated in elderly patients; however, subanalysis of the Candesartan in Heart Failure: Assessment of Reduction in Mortality and Morbidity (CHARM-Alternative) study, with 23.3% of its study population ≥ age 75, demonstrated benefits similar to those reported for the general group.^185^

Regarding beta-blockers, a recent meta-analysis of 12,719 patients did not find any differences in benefits between those defined as "elderly" in the clinical trials included and their younger counterparts. It is important to underline the fact that the oldest patient from the individual clinical trials analyzed was 71 years old.^5^ The Study of Effects of Nebivolol Intervention on Outcomes and Rehospitalization in Seniors with Heart Failure (SENIORS) demonstrated the efficacy of nebivolol in CHF patients > 70 years old. Subanalysis of the Organized Program to Initiate Lifesaving Treatment in Hospitalized Patients with Heart Failure (OPTIMIZE-HF) study provided evidence that beta-blockers may be associated with beneficial effects in patients ≥ 75 years old.^186,187^

The Euro Heart Failure Survey II has suggested that the use of ACEI and/or beta-blockers is associated with a significant decrease in short-term mortality in octogenarians. The Euro HF Survey II, on the other hand, did not show improvements in mortality during 1 year with the use of beta-blockers; this is possibly related to the higher number of elderly HFpEF patients in this study.^188^

In the most important studies with aldosterone antagonists, the Randomized Aldactone Evaluation Study (RALES) and the Eplerenone Post-Acute Myocardial Infarction Heart Failure Efficacy and Survival Study (EPHESUS), the average patient age was 67 and 64, respectively. Their use, in elderly patients, however, should be carefully monitored in accordance with renal dysfunction and the underlying drug interaction. In the Prospective Comparison of ARNI with ACEI to Determine Impact on Global Mortality and Morbidity in Heart Failure (PARADIGM) study, symptomatic hypotension in patients > age 75 was more frequent in the sacubitril/valsartan group (18%) than in the enalapril group (12%).^189^

In summary, current orientations recommend a therapeutic approach similar to the one applied to younger patients for HF treatment, with caution regarding interactions and tolerance.^176,190^

### 4.3. General Recommendations for Elderly Heart Failure Patients

**Table:** 

Complementary diagnostic methods of CHF in elderly patients	Grade of recommendation	Level of evidence
Transthoracic echocardiogram recommended for evaluating structure and function in HF and establishing diagnosis of HFrEF and/or HFpEF	I	C
Transthoracic echocardiogram recommended for evaluating resynchronization/ICD candidates	I	C
Repeat evaluation of ventricular function and measures of structural remodeling in patients with CHF, change in clinical status, or decompensation	I	C
MR with delayed enhancement should be considered in patients with dilated cardiomyopathy to differentiate between ischemic and non-ischemic etiology	IIa	C
MR recommended for cardiac tissue characterization when myocarditis, amyloidosis, sarcoidosis, or non-compacted myocardium are suspected	I	C
Non-invasive stress exams (resonance, echocardiogram, SPECT, PET) are recommended for evaluating myocardial ischemia and viability in patients with CAD and CHF before deciding on revascularization	IIb	B
Cinecoronariography recommended in patients with CHF and angina for diagnosis of CAD	I	C
Coronary angiotomography in patients with CHF and pre-test likelihood indicating low or intermediate risk and in patients whose non-invasive stress exams suggest CAD, with the objective of excluding invasive exams	IIb	C
Hemogram, sodium, potassium, urea, creatinine (clearance), hepatic function, glucose, glycated hemoglobin, TSH, ferritin	I	C
Natriuretic peptides	IIa	C
Electrocardiogram recommended for evaluating rhythm, heart rate, morphology and QRS duration	I	C
Chest radiography recommended to exclude pulmonary alterations. In cases of acute decompensation to detect edema/pulmonary congestion	I	C
Endomyocardial biopsy should be considered for diagnosing specific causes in cases of rapid and progressive worsening in spite of standard therapy	IIa	C

CAD: coronary artery disease; CHF: congestive heart failure; HF: heart failure; HFpEF: heart failure with preserved ejection fraction; HFrEF: heart failure with reduced ejection fraction; ICD: implantable cardioverter-defibrillator; MR: magnetic resonance; PET: positron emission tomography; SPECT: single photon emission computed tomography; TSH: thyroid-stimulating hormone.

**Table:** 

Treatment of comorbidities		
Recommendation	Grade of recommendation	Level of evidence
Iron deficiency – IV iron replacement in patients with ferritin < 100 ng/ml or ferritin between 100 and 199 ng/ml and transferrin saturation < 20% with the objective of improving symptoms and quality of life	IIa	A
Diabetes – metformin use	IIa	C

IV: intravenous.

**Table:** 

Grade of recommendation for pharmacological treatment of HFrEF FC II to IV		
Recommendation	Classification of recommendation	Level of evidence
ACEI in conjunction with beta-blockers with the objective of reducing mortality and hospitalization	I	A
ARB in conjunction with beta-blockers with the objective of reducing hospitalization and mortality in patients with ACEI intolerance	I	B
Addition of aldosterone blockers in symptomatic patients, with LVEF ≤ 35%, associated with ACEI (or ARB) and beta-blockers	I	A
Diuretics to improve symptoms in patients with congestion	I	B
Angiotensin receptor-neprilysin inhibitor (sacubitril/valsartan), to substitute ACEI in order to reduce mortality and hospitalization in patients who continue to be symptomatic in spite of treatment with ACEI (or ARB) and beta-blockers	I	B
Hydralazine and isosorbide dinitrate in African-American patients with EF < 35% or EF < 45% with ventricular dilatation who continue to be symptomatic, with FC III-IV, in spite of treatment with ACEI (or ARB) and beta-blockers to reduce mortality and hospitalization	IIa	B
Hydralazine and isosorbide dinitrate in symptomatic patients with HFrEF who do not tolerate ACEI or ARB to reduce mortality	IIb	B
Digoxin in symptomatic patients with sinus rhythm in spite of treatment with ACEI (or ARB) and beta-blockers to reduce hospitalization	IIb	B
An If channel inhibitor (ivabradine) may be used in symptomatic patients with sinus rhythm, EF < 35%, and HR > 70 bpm, in spite of treatment with ACEI (or ARB) and beta-blockers to reduce hospitalization and mortality	IIa	B

ACEI: angiotensin converting enzyme inhibitors; ARB: angiotensin receptor blockers; EF: ejection fraction; FC: New York Heart Association functional class; HFrEF: heart failure with reduced ejection fraction; HR: heart rate; LVEF: left ventricular ejection fraction.

**Table:** 

Available drugs, initial and target doses, dose adjustments, and safety in elderly patients				
Drugs	Initial dose	Maximum dose	Dose adjustment for elderly patients	Safety in elderly patients
Captopril	6.25 mg 3×/day	50 mg 3×/day	None	Increase in orthostatic hypotension Take before bedtime Decrease diuretics
Enalapril	2.5 mg 2×/day	10–20 mg 2×/day	None	More susceptible to renal dysfunction
Lisinopril	2.5–5.0 mg 1×/day	20–40 mg 1×/day	None	Avoid use of NHAI drugs
Perindopril	2.0 mg 1×/day	8,0–16 mg 1×/day	None	
Ramipril	1.25–2.5 mg 1×/day	10 mg 1×/day	Adjust according to renal function	
Candesartan	4.0–8.0 mg 1×/day	32 mg	None, but elevated AUC and Cmax	Similar to that of ACEI
Losartan	25 mg 1×/day	50–100 mg	None	
Valsartan	40 mg 2×/day	320 mg	None	
Bisoprolol	1.25 mg 1×/day	10 mg 1×/day		Water retention: - Monitor weight daily - Adjust diuretic dosage Risk of hypotension and bradycardia: - Start with a low dose and increase progressively - Adequate hydration Increased fatigue: - Improves over time - Consider comorbidities anemia
Carvedilol	3.12 5mg 2×/day	50 mg/day	None	
Metoprolol succinate	12.5–25 mg	200 mg/day	None	
Nebivolol	1.25 mg	10 mg	None	
Spironolactone	12.5–25 mg	25–50 mg	None	Increased risk of hyperkalemia and renal dysfunction Monitor K and creatinine
Furosemide	20–40 mg/day 1 or 2×/day	600 mg (usual 40–240 mg/day)	Start 20 mg/day	Frequent monitoring Increased risk of alterations in water balance and electrolyte disturbances
Bumetanide	0.5–1 mg 1 or 2×/day	10 mg Usual (1–5 mg/day)	None	Frequent monitoring Increased risk of alterations in water balance and electrolyte disturbances
Hydrochlorothiazide	25 mg	200 mg/day Usual (12.5–100 mg/day)	Start 12.5 mg–25 mg	Monitor fluid volume and electrolyte status
Chlorthalidone	12.5–25 mg	100 mg	None	Monitor fluid volume and electrolyte status

ACEI: angiotensin converting enzyme inhibitors; AUC: area under curve; NHAI: non-hormonal anti-inflammatory.

## 5. Arterial Hypertension in The Elderly

### 5.1. Diagnostic Peculiarities

A Brazilian epidemiological study titled the Multicenter Study of Elderly Patients in Outpatient Clinics of Cardiology and Geriatric Brazilian Institutions (EMI, acronym in Portuguese)^191^ demonstrated that SAH is the main risk factor among elderly Brazilians. It is found in 65% of elderly outpatients and 80% of women > 75 years old. Aging produces vascular alterations, such as arterial stiffening, reduced elasticity and vascular compliance, reduced vasodilation capacity, increased SBP, decreased sensitivity to volume changes, slowed ventricular relaxation, increased cardiac workload, loss of myocytes, and compensatory hypertrophy.^192^ These alterations lead to peculiarities in diagnosing and treating SAH in elderly patients.

#### 5.1.1. Peculiarities in Measuring Blood Pressure

In elderly patients, BP has high variability. It is necessary to take special care in measuring BP, owing to the possible presence of the following factors:

a) OH: defined as a drop in SBP of > 20 mmHg or in diastolic blood pressure (DBP) of > 10 mmHg, following 3 minutes in the orthostatic position. BP should be checked in the sitting, lying, and standing positions, given that atherosclerotic alterations in the carotid sinus regions may reduce baroreceptor sensitivity, leading to reduced postural reflexes and, thus, predisposing the patient to OH.^3^ Furthermore, comorbidities, such as peripheral polyneuropathy and Parkinson's disease, as well as the use of diuretic, antidepressant, vasodilator, and beta-blocker drugs may also lead to OH in up to 34% of elderly patients > age 75.
b) Auscultatory gap: a sitution in which, after auscultation of the first Korotkoff sound, the sound disappears completely and only reappears after the decrease in SBP, but before the beginning of the last phase of Korotkoff sounds. This leads to errors in diagnosing SBP at lower levels and false diagnoses of normotension. In order to avoid this measurement error, it is necessary to estimate systolic pressure using the radial pulse palpatory method, raising cuff pressure 20 to 30 mmHg above this point.^193^
c) Pseudo-hypertension: pseudo-hypertension may appear in elderly patients with pronounced atherosclerosis, arterial wall calcification, and vessel stiffening. In this situation, it is sufficient to inflate the cuff in order to collapse the brachial artery.^193^ Osler's maneuver is used to identify this. The maneuver consists of inflating the cuff above systolic pressure levels and, concomitantly, palpating the radial artery. If it continues to be palpable, this suggests that the artery is stiff and indicates that the index obtained by auscultation does not express the true SBP. Pseudo-arterial hypertension may also be suspected when SBP is elevated in patients who do not present injuries in target organs or in those who manifest hypotension following treatment with low doses of anti-hypertensive drugs.
d) Arterial hypertension during exercise: although BP is habitually higher during physical exercise, this increase is greater in elderly adults, due to arterial stiffness. Values for diagnosing SAH during exercise are not clear. Physically deconditioned patients respond with greater increases in BP than conditioned patients.
e) White coat hypertension: this occurs when BP increases during a clinical visit but remains normal during daily activity. This can be better evaluated by 24-hour ambulatory blood pressure monitoring (ABPM) or home blood pressure monitoring (HBPM).^193^ Serial measurements may minimize this condition.
f) Masked arterial hypertension: this is the opposite of white coat hypertension, namely, pressure is high during daily activities and normal during the clinical visit.^193^ This may also be evaluated by 24-h ABPM or HBPM.
g) Isolated systolic hypertension (ISH) and pulse pressure (PP): ISH and PP are cardiovascular risk factors in elderly patients.^191^ ISH is due to lower distensibility and elasticity in the large capacitance vessels, such as the aorta, which results in increased pulse wave velocity (PWV). This increase in PWV is accompanied by an increase in reflex wave velocity, which returns from peripheral to central circulation.^191,192^ In elderly patients, the reflex wave reaches the ascending aorta during systole, leading to an even higher increase in SBP. Loss of reflex wave in protodiastole makes diastolic pressure remain equal or decrease.^192^ The final effect consists of a predominant increase in SBP, with DBP remaining normal or even low. Characteristics of ISH include SBP ≥ 140 mmHg and DBP < 90 mmHg.^193^ PP is defined as the difference between SBP and DBP. This occurs due to the progressive loss of arterial elasticity, with a consequent decrease in vascular complacency. DBP tends to remain normal or even low. Limits for abnormal PP values have yet to be defined.^191^ The Framingham study demonstrated a higher cardiovascular risk associated with higher PP, in patients between the ages of 50 and 79, as well as an important role of low DBP in this association.^3^ In addition to the factors mentioned, target organ injuries should be investigated (eye fundus changes, LV hypertrophy, and peripheral and renal atherosclerosis), and the possibility of secondary SAH should be evaluated. The following are suspicious factors:^193^
  a) Sudden onset of SAH or acute worsening
  b) Abdominal murmur
  c) SAH resistant to 3 or more drugs
  d) Creatinine increase over 30% with the use of ACEI or ARB
  e) Systemic atherosclerotic disease in smokers and patients with dyslipidemia
  f) Recurrent hypertensive pulmonary edema
  g) Pheochromocytoma and hyperaldosteronism should be adequately investigated with more specific exams, because, even though they are less frequent in elderly patients, once they are diagnosed and treated, they may result in the patient being cured.

Among secondary causes of SAH, the following stand out: aortic regurgitation (AR), hyperthyroidism, renovascular atherosclerosis, and use of drugs that increase pressure, such as non-hormonal anti-inflammatory agents, antihistamines, decongestants, corticosteroides, MAOI, and TCA.

#### 5.1.2. Peculiarities of Clinical Laboratory Investigation

The objective of clinical laboratory investigation is to confirm that BP is increased; identify causes of SAH, target organ injuries, and associated diseases; and stratify cardiovascular risk. In addition to clinical history, cognitive tests, and physical examination including BMI and abdominal circumference, the following should be performed:

a) Resting EKG.
b) Urine examination (biochemical and sediment)
c) Blood tests: complete blood count, creatinine, blood glucose, potassium, fasting blood glucose, glycohemoglobin, total cholesterol and fractions, triglycerides, and uric acid. Blood levels of creatinine may be normal, in spite of declined renal function. This fact results from the progressive loss of muscle mass, a determining factor of creatinine production. Thus, creatinine levels > 1.5 mg/dL are considered abnormal in elderly patients. The formula most used to calculate estimated glomerular filtration rate (eGFR) is the Cockroff-Gault (mL/min): (140 - age) × weight (kg)/plasma creatinine (mg/dL) × 72, with a coefficient of 0.85 for women. Interpretation: normal renal function, > 90 mL/min; slight renal dysfunction, 60 to 90 mL/min; moderate renal dysfunction, 30 to 60 mL/min; severe renal dysfunction, < 30 mL/min.
d) ABPM and HBPM: to investigate white coat SAH and masked SAH, in cases where it is necessary to investigate episodes of arterial hypotension, or to evaluate the efficacy of SAH therapy.^193^

### 5.2. Treatment Peculiarities

#### 5.2.1. Therapeutic Goals for Elderly Patients

Treating SAH in elderly patients represents a great challenge, as it involves a heterogeneous group, with multiple comorbidities, cognitive problems, risk of falling, polypharmacy, and frailty syndrome. Therapeutic goals for elderly patients should thus be individualized based on multidisciplinary team judgment, and they should consider patient preferences.^193,194^ Dose adjustments should occur every 4 weeks, in order to avoid abrupt reductions of BP. The Hypertension in the Very Elderly Trial (HYVET) randomized, placebo-controlled study^194^ included 3,845 patients with SBP ≥ 160 mmHg over the age of 80, with an average age of 83.6. Target blood pressure was 150/80 mmHg. They demonstrated that treatment with indapamide, with or without perindopril, was beneficial in octogenarians. In the intention-to-treat analysis, there was a 30% reduction in rate of fatal or non-fatal stroke, 39% reduction in the rate of death from stroke, a 21% reduction in death from any cause, a 23% reduction of in the rate of death from cardiovascular causes, and a 64% reduction in the rate of HF. Fewer severe adverse events occurred in the active treatment group (358 versus 448 in the placebo group). There is evidence that greatly lowering BP in elderly patients may be harmful; this fact is known as the J- or U-curve.^191^ The recent SPRINT study^149^ sought to evaluate two different BP goals. In the standard group, the goal was SBP < 140 mmHg and, in the intensive treatment group, the goal was SBP < 120 mmHg. The intensive treatment group had a significant reduction in primary events (infarction, other acute coronary syndromes, stroke, HF, or death from cardiovascular causes) in comparison with the standard treatment group. Although the initial impression may be that more intensive goals may be more beneficial, it is necessary to consider that there was an increase in the number of severe adverse events, such as hypotension, syncope, electrolytic disorders, and acute renal insufficiency, in the intensive treatment group. Another important study was the ACCORD,^35^ performed with 10,251 diabetic patients, ages 40 to 79, 4,733 of which were also randomized for BP reduction < 140 mmHg or < 120 mmHg. However, BP reduction with more intensive goals did not succeed in significantly reducing the risk of the study's primary outcome (death from CVD, nonfatal infarction, and nonfatal stroke). Thus, to date, the III Geriatric Cardiology Guidelines recommends SBP levels ≤ 130 mmHg for elderly patients ≥ age 65, who are considered robust and who do not have frailty criteria.^195,196^ For patients ≤ 80 years old, without frailty, SBP levels < 140 mmHg may be considered;^195^ in patients ≥ age 80 with SBP ≥ 160 mmHg, an initial reduction to SBP between 150 and 140 mmHg may be considered;^7^ in fragile elderly patients or patients with multiple comorbidities, the therapeutic goal should be individualized, considering each case's risk-benefit ratios.^196^

#### 5.2.2. Medical and Non-Medical Treatments

Salt reduction should be cautious and well accompanied by the doctor, given that the elderly patient's diminished taste sensitivity may make food appear blander, causing the patient to eat less and thus bringing about the risk of malnutrition. It is also necessary to remember that elderly patients rarely present only one chronic disease. The evaluation of multimorbidities generally defines what the best treatment is and what drugs should be avoided in each specific case. Treatment should be initiated with low doses, and dose adjustments should be gradual. Adherence needs to be stimulated, if possible, by monthly control at the beginning of treatment and at each dose adjustment. The most commonly used drugs in elderly patients are:

a) Diuretics: thiazides and correlates (hydrochlorothiazide, chlorthalidone, indapamide) are considered first-line drugs in elderly patients without comorbidities. Their use is preferential in osteoporosis patients, as they decrease urinary excretion of calcium, and in initial phases of congestive heart failure (CHF), as they reduce preload, volume, and pulmonary congestion. Recommended doses of hydrochlorothiazide: 6.25 to 25 mg/day, maintaining efficacy and reducing adverse metabolic effects.^191^ In most cases, diuretics are associated with therapeutic schedules. However, they should be avoided in elderly patients with incipient urinary incontinence, gout (because they increase uric acid) and prostatism.^191^ Attention should be paid to blood glucose in elderly patients with concomitant use of thiazides and oral antidiabetics or insulin, given that thiazides may increase blood glucose and interfere with diabetes control.
b) Calcium channel blockers: they include both dihydropyridine and non-dihydropyridine derivatives. Dihydropyridine derivatives have major vasodilatory effects. The most recent generation provokes less edema. They are very commonly used in elderly SAH and symptomatic coronary disease patients. Non-dihydropyridine derivatives, especially verapamil, have fewer vasodilatory effects, and they are not usually prescribed to elderly patients, as they may alter electrical impulses of atrioventricular conduction. Verapamil may, furthermore, provoke intestinal constipation.
c) ACEI: they continue to be efficacious in elderly patients, notwithstanding the decrease in renin with aging. They decrease cardiovascular events and should be used in elderly patients with SAH and HF or asymptomatic ventricular dysfunction. Adverse effects include changes in taste, especially with captopril, which may reduce food intake, and dry cough, which limit their use. It is fundamental to check potassium, due to frequent reductions in renal function.
d) Angiotensin II receptor antagonists (ARA-II): they are effective in HF, and they have an established renal and cardiac protective action in type 2 diabetes with nefropathy.^191^ ARA-II have a favorable tolerability profile, with few adverse effects (occasional dizziness and, rarely, hypersensitive skin reaction). They are well used in cases of ACEI intolerance.^193^
e) Beta-blockers: they are not used as initial monotherapy in elderly patients without comorbidities, due to their lower effects on BP reduction; however, in association with diuretics, they present good results. They are mainly used in elderly patients with SAH and coronary insufficiency or HF. Less liposoluble beta-blockers, such as atenolol, metoprolol, and bisoprolol, are recommended for elderly patients because they have lower risks of collateral effects on the central nervous system (depression, drowsiness, confusion, sleep disturbances).^193^

In summary, elderly patients have particularities regarding SAH diagnosis and approach. It is necessary to consider each patient's comorbidities and particularities, including functional status, which may be determining factors for setting BP goals and for patient decision making.

**Table:** 

Recommendation	Grade of recommendation	Level of evidence
SBP ≤ 130 mmHg for elderly patients ≥ age 65, without frailty	I	A
SBP < 140 mmHg for elderly patients ≤ age 80, without frailty	IIb	C
For elderly patients > age 80, with initial SBP ≥ 160 mmHg, initial SBP reduction between 150 and 140 mmHg	I	B
In fragile elderly patients or patients with multiple comorbidities, the therapeutic goal should be individualized, considering risk-benefit ratios	IIa	C

SBP: systolic blood pressure.

## 6. Valvulopathies

### 6.1. Mitral Stenosis

#### 6.1.1. Diagnostic Peculiarities

Mitral stenosis (MS) is rare in elderly patients (present in 6% of patients with mitral annulus calcification).^197^

Etiology - Sequel of rheumatic carditis or calcification of the mitral valve apparatus in patients > 85 years old.^198^

Symptoms - similar to those observed in non-elderly patients. Symptoms may be absent. The most frequent are dyspnea and cough, which may be accompanied by hemoptoic sputum. It may manifest as systemic embolism or AF.

Physical examination - Hyperphonetic first heart sound and apical mid-diastolic murmur with thrill may be absent. The opening snap of the mitral valve is rarely auscultated. Most patients > age 80 present AF with elevated HR which, in association with a greater anteroposterior thorax diameter, makes auscultation difficult. The more fibrosis and calcification are present in the mitral valve, the less audible the auscultatory signs of MS will be. Diagnostic suspicion may be established based on signs of pulmonary arterial hypertension (P2 hyperphonesis in the second heart sound, RV insufficiency, pulmonary and tricuspid regurgitation). In elderly pulmonary arterial hypertension patients without any other evident cause, it is important to investigate MS.^199-202^

Complementary exams - EKG, chest radiography, and echocardiogram are sufficient, in most cases, to confirm diagnosis and estimate severity. The following may be found in the EKG: left atrial overload (LAO), RV hypertrophy, and AF. Chest radiography findings include: increased LA, mitral valve calcification, and posterior displacement of the barium-filled esophagus. Echocardiography data include: mitral annulus calcification (in 60% of elderly patients > age 85),^4^ mitral valve area (Table 8), pulmonary BP, and status of the valvular apparatus (mobility, thickening, and subvalvular impairment).^5^

**Table 8:** Severity of mitral stenosis

	Pressure gradient (LA-LV) in mmHg	Mitral valve area (cm²)
Mild	< 5	> 1.5
Moderate	5 a 10	1 a 1.5
Severe	> 10	< 1

LA: left atrium. LV: left ventricle.^202^

#### 6.1.2. Treatment Peculiarities

Clinical treatment - Patients with mild MS are generally asymptomatic, and they do not need to receive medication,^203^ unless they also suffer from AF. Unlike younger patients, elderly MS patients who develop AF have a higher chance of showing symptoms of HF, owing to the concomitant present of diastolic dysfunction. Thus, in cases of paroxysmal AF with hemodynamic deterioration, even if MS is mild, electrical cardioversion is indicated. Patients with MS and AF, be it permanent, persistent, or paroxysmal, should constantly use warfarin, regardless of risk scores, with the aim of keeping the international normalized ratio (INR) between 2 and 3, unless there is a formal contraindication.^204^ Although some publications recommend the use of new oral anticoagulants in this situation, these data have yet to be evaluated in comparative studies.^205^ The finding of LA thrombus or the occurrence of a systemic embolic event, even in the presence sinus rhythm (SR), also indicate the need for anticoagulant use. In MS of rheumatic etiology, prophylaxis for rheumatic fever is not necessary, given that elderly patients rarely have relapses of this disease.^206^ Early treatment of bacterial infections is recommended with the aim of protecting the patient from the risk of infective endocarditis (IE). Chemoprophylaxis against IE in elderly MS patients is not indicated.^207^ In symptomatic patients with moderate to severe MS, loop diuretics are the best option for controlling pulmonary or systemic congestion, and beta-blockers are indicated for reducing HR and facilitating atrial emptying. There is no evidence that the use of beta-blockers is beneficial in patients with SR who do not have elevated HR.^208^ In the presence of AF with elevated ventricular response, beta-blockers are the drugs of choice for reducing HR. In cases where they are contraindicated, nondihydropyridine calcium channel blockers or digitalis may be used. In the presence of signs of RV failure with associated hepatomegaly, due to the frequent coexistence of secondary hyperaldosteronism, elevated doses of spironolactone (100 mg/day) are an option.^209^ Caution is necessary with the risk of hyperkalemia.

Options for correcting MS - When evaluating an elderly patient with MS who has been indicated for intervention, the following should be considered and discussed with the patient and/or family members: etiology, whether rheumatic or degenerative; patient life expectancy; evaluation of functionality; and the presence of multimorbidities. There are 2 options for correcting rheumatic MS: percutaneous balloon mitral valvuloplasty (PBMV) or extracorporeal circulation surgery. Randomized clinical trials have shown that, in selected cases, PBMV offers immediate and long-term results similar to those of open commissurotomy.^210^ For this intervention, presence of favorable valve morphology is important. This may be evaluated by several proposed echocardiography criteria, the Wilkins and Block score being the most widely used.^211^ It is, additionally, necessary to respect contraindications to this procedure (presence of LA thrombus or mitral regurgitation with more than a mild degree of severity). Unfortunately, elderly patients frequently have valve morphologies which are unfavorable for this procedure, whether the etiology be rheumatic or degenerative.^212^ In the latter case, owing to the fact there is no commissural fusion, as occurs in rheumatic disease, the success of PBMV is restricted, and mitral valve replacement surgery is the procedure of choice. As degenerative MS patients frequently have multimorbidities that elevate their risks, clinical treatment should be attempted initially; mitral valve replacement surgery is indicated only in cases that do not respond to clinical treatment.^213^ There are reports of small series of percutaneous implants of mitral prostheses in degenerative MS patients, with relative success.^214^

**Table:** 

Medical treatment of elderly mitral stenosis patients		
Recommendation	Grade of recommendation	Level of evidence
Regardless of severity, MS patients who have AF, be it permanent, persistent, or paroxysmal, should receive warfarin indefinitely, with the aim of keeping INR between 2 and 3, unless this is contraindicated	I	B
MS patients indicated for warfarin may use direct oral anticoagulants	IIb	C
Elderly rheumatic MS patients should receive prophylaxis to prevent rheumatic fever	III	C
Elderly MS patients with MVA less than or equal to 1.5 cm^2^; FC II, III, or IV; and/or signs of RVF should receive loop diuretics to alleviate symptoms	I	C
Elderly MS patients with MVA less than or equal to 1.5 cm^2^; FC II, III, or IV; and SR, who continue to be symptomatic in spite of diuretic use, if HR is over 60 bpm, should receive beta-blockers, unless there are contraindications	IIa	B
Elderly patients with mild MS who develop AF with elevated ventricular response should receive beta-blockers to control ventricular response, unless there are contraindications	IIa	C
In the previously described cases, nondihydropyridine calcium channel blockers or digitalis may be used, in the event that beta-blockers are contraindicated	IIa	C
MS patients with signs of RVF and hepatomegaly, without adequate response to loop diuretics, should receive spironolactone.	IIb	C

AF: atrial fibrillation; bpm: beats per minute; FC: New York Heart Association functional class; HR: heart rate; INR: international normalized ratio; MS: mitral stenosis; MVA: mitral valve area; RVF: right ventricle failure; SR: sinus rhythm.

**Table:** 

Indications for intervention in elderly rheumatic mitral stenosis patients		
Recommendation	Grade of recommendation	Level of evidence
Elderly symptomatic rheumatic MS patients (FC II-IV), with MVA ≤ 1.5 cm^2^, who have favorable valve morphology and no contraindications, should undergo PBMV	I	A
Elderly rheumatic MS patients who, although they are very symptomatic (FC III/IV) with MVA ≤ 1.5 cm^2^, but with unfavorable valve morphology or contraindication to PBMV, without elevated surgical risk or low life expectancy, should be referred for open valvuloplasty or valve replacement surgery	I	B
If the MS patient is in FC II with MVA ≤ 1.5 cm^2^, but is not a candidate for PBMV, it is prudent to maintain medical treatment as long as the patient does not become more symptomatic	IIb	C
Rheumatic MS patients with MVA ≤ 1.5 cm^2^ who are indicated for AVR, ascending aorta surgery, or MRS, should also undergo valvuloplasty or mitral valve replacement	I	C
PBMV is indicated for rheumatic MS patients, with MVA ≤ 1.5 cm^2^, even if they are asymptomatic, notwithstanding pulmonary arterial hypertension (PASP > 50 mmHg), whose probably etiology is MS, when valve morphology is favorable, in the absence of contraindication	IIa	C
Severe rheumatic MS patients (MVA ≤ 1.0 cm^2^), who are asymptomatic and who have favorable valve morphology for PBMV and no contraindications, should undergo the procedure	IIb	C

AVR: aortic valve replacement; FC: New York Heart Association functional class; MS: mitral stenosis; MRS: myocardial revascularization surgery; MVA: mitral valve area; PASP: pulmonary artery systolic pressure; PBMV: percutaneous balloon mitral valvuloplasty.

**Table:** 

Indications for intervention in elderly degenerative mitral stenosis patients		
Recommendation	Grade of recommendation	Level of evidence
MVR in elderly degenerative MS patients who do not respond adequately to clinical treatment and who have low surgical risk and high life expectancy	IIa	C
PBMV in elderly degenerative MS patients, FC III/IV, who do not respond to clinical treatment, with high surgical risk	IIb	C
Percutaneous implants of mitral prosthesis in very symptomatic patients who do not respond to clinical treatment and who are not candidates for open surgery or PBMV	IIb	C

FC: New York Heart Association functional class; MS: mitral stenosis; MVR: mitral valve replacement; PBMV: percutaneous balloon mitral valvuloplasty.

### 6.2. Mitral Regurgitation

#### 6.2.1. Diagnostic Peculiarities

From the etiological point of view, mitral regurgitation (MR) may be: (a) primary: when there are histological changes in the valve, for example, myxomatous degeneration, degenerative fibroelastic disease, and IE; or (b) secondary: when MR is functional and the valve is histologically normal, for example, poor leaflet coaptation with dilated cardiomyopathy. MR is common in elderly patients; the degenerative cause is the most frequent, followed by ischemia, and, less frequently, rheumatic disease and IE.^215,216^ Acute MR is mainly linked to CAD by papillary muscle dysfunction or chordae tendineae rupture, with condition of acute HF.

Symptoms - symptoms of chronic MR are related to severity, rate of disease progression, pulmonary BP, presence of arrhythmias (e.g., AF), and associated diseases. The most common symptoms are stress dyspnea and fatigue.

Physical examination - The following are present: protosystolic murmur in mitral focus, variable intensity, and displaced ictus, with characteristics of volumetric overload. Thoracic deformities, which are common at this age, may modify ictus, sounds, and murmurs.^102,202^

Complementary exams - During EKG, frequent abnormalities are LAO, AF and left ventricular overload (LVO).^217^ In the presence of ischemic MR, electrocardiographic signs of coronary insufficiency, such as electrically inactive zones and alterations in ventricular repolarization, may occur.^218^ In cases of acute MR, EKG may be normal, or it may show only sinus tachycardia.^217,219^ Chest radiography aids detection of comorbidities, evaluation of pulmonary congestion, and distinction between acute and chronic cases. In cases of acute MR, the heart may have normal dimensions, and pulmonary congestion may, nevertheless, be present. In cases of chronic MR, there will be an increase in the LA and LV.^217,218,220^ Transthoracic echocardiography is indispensable for diagnosing and evaluating degree of mitral regurgitation, chamber size, and ventricular function. The sizes of the LA and LV and measurements of pulmonary artery pressure are especially important. Identification of the cause and detailed evaluation of valvular apparatus impairment, leaflet morphology, and reflux mechanism are important for deciding whether the most adequate treatment is mitral valve replacement or plasty.^202,221,222^ Transesophageal echocardiography (TEE) may be used when there are technical difficulties to acquiring an adequate echocardiography window. Cardiac catheters are indicated for diagnosis of CAD in patients referred for surgery and in cases where there are doubts regarding the severity of the lesion.^102,202,221,222^ ET/ergospirometry may be used to evaluate the reproduction of symptoms and changes in tolerance to exercise. They are less used with very elderly patients with physical limitations.^202,217,221,222^ Magnetic resonance and computerized tomography are not routinely used in patients with mitral disease, but they may be indicated when the severity of MR or LV function have not been adequately evaluated by echocardiogram or when there are discrepancies.^221,222^

#### 6.2.2. Treatment Peculiarities

Treatment of MR should consider its etiology and severity. AF, pulmonary hypertension, and symptoms are relevant factors in the decision making process. Elderly patients > age 75 have elevated surgical risks. Surgical management in this age range will aim to improve and maintain quality of life. Thus, the symptoms present are a determining factor for surgical indication. Patients with ventricular dysfunction who are asymptomatic should continue clinical treatment.^221^ Therapeutic decisions for MR should be guided by presentation (acute or chronic), clinical hemodynamic profile, and severity of symptoms. Echocardiography parameters, such as LVEF, left ventricular end-systolic diameter (LVESD), and the presence of dyspnea are indicators for surgical therapy (See the following recommendations table). Mitral plasty is the preferred surgical treatment. Currently, mitral clips are an incipient and promising alternative.^221,223^

Treatment of acute MR - In patients with acute, severe MR, immediate surgical treatment is recommended. Some patients with moderate MR may develop hemodynamic compensation due to LV dilation, thus making lower filling pressure and normalization of cardiac output possible. In cases of chordae tendineae rupture, mitral repair is preferable to mitral replacement, and surgery may be scheduled according to the patient's clinical and hemodynamic status.^221,223,224^ Medical treatment of acute MR must by implemented as a support therapy for the definitive surgical correction.^221^ In the presence of severe manifestations, such as acute pulmonary edema or shock, vasoactive drugs, such as intravenous vasodilators, sodium nitroprusside, nitroglycerin, and vasopressin amines, in addition to an intra-aortic balloon for hemodynamic support, should be used up to the moment of the indicated surgical procedure.^223^

Treatment of chronic MR - Patients with chronic, asymptomatic MR and normal LVEF are not indicated for medical treatment. There is no evidence that long-term treatment with vasodilators presents therapeutic benefits.^221^ In symptomatic patients, treatment with ACEI, beta-blockers, such as carvedilol, and diuretics should be implemented.^224,225^ Biventricular pacemakers in patients classified as "responders" show improvements in MR in reverse LV geometry.^226^ Patients with symptomatic chronic primary MR should undergo surgical treatment, preferably plasty, regardless of LV function. Asymptomatic patients who have progressive dysfunction (LVEF < 0.60) and/or increased ventricular diameters (LVESD > 45 mm), should also be considered surgery. Indication for valve surgery in elderly patients > age 75 has not been consistently evaluated in clinical trials, it being necessary to prioritize the presence of symptoms as an indication for invasive intervention. In valve replacement surgery, bioprostheses are indicated in elderly patients owing to their lower rates of prosthetic dysfunction and to the inherent risks of anticoagulant therapy.^227,228^

Percutaneous treatment of mitral regurgitation - Percutaneous treatment of MR has been performed, particularly in Europe. In Brazil, MitraClip® is the only commercially available device, and it is used only in select cases, owing to the high cost. The use of this device is indicated in patients whose primary chronic MR is degenerative in etiology and whose surgical risks are high or prohibitive. Furthermore, patients with chronic MR secondary to ventricular dilation who are refractory to optimized clinical treatment and cardiac resynchronization may eventually benefit from this procedure. In symptomatic patients with severe MR due to degeneration of a bioprosthesis or previously implanted valve rings and prohibitive surgical risks, percutaneous mitral replacement via the valve-in-valve procedure at a specialized center is an alternative. Percutaneous mitral replacement for symptomatic patients with severe native valve MR and prohibitive surgical risks is at an advanced phase of development and should be available in Brazil in the coming years.^229^

**Table:** 

Recommendations for MR surgery		
Recommendation	Grade of recommendation	Level of evidence
Symptomatic patients with severe acute MR	I	C
Symptomatic patients with severe chronic primary MR and normal left ventricular function	I	B
Asymptomatic patients severe chronic primary MR and left ventricular function (EF 30-60% and/or end-systolic diameter ≥ 40 mm)	I	B
Plasty is preferable to mitral replacement in severe chronic primary MR patients	I	B
Plasty or mitral replacement is indicated in patients with severe chronic primary MR and patients undergoing concomitant heart surgery	I	B
Mitral replacement is preferable to plasty in patients with chronic secondary MR of ischemic etiology	I	A
Mitral plasty may be considered for chronic primary (non-rheumatic) MR, normal ventricular function, and new atrial fibrillation or pulmonary hypertension (resting PASP > 50 mmHg)	IIa	B
Plasty or mitral replacement may be considered for symptomatic patients with chronic primary MR and FE ≤ 30%	IIb	C
Mitral plasty via catheter may be considered for symptomatic patients (FC III/IV) with chronic primary MR and prohibitive surgical risk	IIb	B
Mitral plasty may be considered for symptomatic patients (FC III/IV) with chronic secondary (functional) MR who are refractory to clinical treatment and cardiac resynchronization	IIb	C
For symptomatic patients with severe MR due to degeneration of a bioprosthesis or previously implanted valve rings and prohibitive surgical risks, percutaneous mitral replacement at a specialized center may be considered	IIb	C
Asymptomatic patients with severe MR and preserved left ventricular function (LVEF > 60% and end systolic diameter < 40 mm)	III	C
Plasty or mitral replacement may be considered for patients with moderate MR who are undergoing concomitant myocardial revascularization surgery	III	A

EF: ejection fraction; FC: New York Heart Association functional class; LVEF: left ventricular ejection fraction; MR: mitral regurgitation; PASP: pulmonary artery systolic pressure.

### 6.3. Aortic Stenosis

#### 6.3.1. Diagnostic Peculiarities

In order to diagnose AS, the most frequent valvulopathy in elderly patients, it is necessary to consider clinical history, which may be difficult in this age range due to possible cognitive and sensory alterations.

Symptoms - Patients may be asymptomatic or present dyspnea, angina pectoris, or syncope.

Physical examination - Findings may include: (a) impulsive type *ictus cordis*, which may be absent in elderly patients due to increased anteroposterior diameter of the rib cage; (b) the *parvus et tardus* pulse (reduced amplitude and longer duration time), which is characteristic of AS in younger patients, may be absent in elderly patients, due to the stiffening of arterial walls which promotes an increase in PWV, thus masking this semiological finding; (c) mid-systolic murmur in crescendo and decrescendo which radiates toward the neck and clavicles. Gallavardin's phenomenon is frequently auscultated. This is a radiation of the AS murmur to the apical region; (d) hypophonetic second sound.

Complementary exams - EKG may present findings compatible with LAO and LVO. Chest radiography may be normal, in approximately half of elderly patients examined, or there may be aspects of hypertrophy, which may or may not present post-stenotic aortic dilatation. Echocardiography is a fundamental exam for diagnosing and classifying this valvulopathy. Three echocardiography parameters are frequently used to classify severity of AS: (a) peak aortic jet velocity; (b) mean transvalvular gradient; (c) valve area (Table 9). The ET has been indicated for asymptomatic patients with severe AS in order to verify the hemodynamic response to effort; on the other hand, its use in elderly patients should be individualized, owing to the presence of multimorbidities which may impede the procedure.

**Table 9:** Diagnosis and classification of aortic stenosis severity

Indicator	Mild	Moderate	Severe
Jet velocity (m/s)	< 3.0	3.0 to 4.0	> 4.0
Mean gradient (mmHg)	< 25	25 to 40	> 40
Valve area (cm^2^)	> 1.5	1.0 to 1.5	< 1.0

#### 6.3.2. Treatment Peculiarities

Medical treatment - Arterial hypertension is common in elderly AS patients. It contributes to increased total afterload, in conjunction with obstruction, thus promoting LV overload. In elderly patients, it is necessary to begin antihypertensive treatment with low doses and gradually increase posology. It is necessary to be cautious when using diuretics, due to the risk of hypotension. ACEI may be advantageous due to their effect on ventricular fibrosis, and beta-blockers are appropriate in patients with CAD. Statin use is not indicated for preventing the progression of AS.^203^ In the presence of HF, beta-blockers should be initiated with low doses, and the same precautions should be taken in prescribing aldosterone antagonists, ACEI, and ARB, and especially with digitalis drugs, as their toxicity and therapeutic thresholds are close.^203^ In elderly patients, it is important to evaluate creatinine clearance in order to adjust dosages and thus avoid drug intoxication.

Surgical treatment - Indicating surgery, whether aortic valve replacement or transcatheter aortic valve implantation (TAVI), depends on a set of factors, including: severity of valve lesion; complementary exam data; evaluation of multimorbidities; risk scores, for example the STS score; and functional evaluation (frailty and cognitive function). Deciding on percutaneous implantation requires a multidisciplinary team for integrated action.^203,230^ The first step in deciding on surgery is establishing that the patient has a severe aortic valve lesion, which, associated with the presence of symptoms, presents a high grade of recommendation. Surgical treatment may still be offered to asymptomatic patients with ventricular dysfunction (LVEF < 50%) or who have already scheduled another cardiac surgery.^203^ In relation to the risks of surgical procedures, patients are classified as low risk: STS < 4%, without frailty, without comorbidity; intermediate risk: STS 4% to 8%, mild frailty, affected organic system; high risk: STS > 8%, moderate to severe frailty, more than 2 affected organic systems; prohibitive risk: pre-operative risk > 50% in 1 year, 3 affected organic systems, or extreme frailty.^203,231^ In most cases, the decision is complex, making it necessary to involve family and the medical and multidisciplinary team and, above all, to respect the patient's own wishes. When the benefits are considered less than the risks, palliative care may be the patient's best option.

**Table:** 

Recommendations for medical treatment of AS		
Recommendation	Grade of recommendation	Level of evidence
Systemic arterial hypertension should be treated in asymptomatic AS patients, starting with a low dose of anti-hypertensive and gradually increasing, as necessary, with frequent clinical follow-up	I	B
Vasodilator therapy may be used in association with invasive hemodynamic monitoring to treat patients with severe decompensated AS, with New York Heart Association class IV symptoms of HF	IIb	C
Statin use is not indicated to prevent the progression of AS in patients with mild to moderate calcified lesions	III	A

AS: aortic stenosis, HF: heart failure.

**Table:** 

Recommendations for surgical treatment of AS		
Recommendation	Grade of recommendation	Level of evidence
Symptomatic patients with severe AS	I	B
Asymptomatic patients with severe AS and LVEF < 50%	I	B
Patients with severe AS scheduled to undergo other cardiac surgeries	I	B
Asymptomatic patients with very severe AS (transvalvular jet velocity ≥ 5.0 m/s) and low surgical risk	IIa	B
Asymptomatic patients with severe AS and diminished exercise tolerance or	IIa	B
effort hypotension	IIa	C
Patients with moderate AS scheduled to undergo other cardiac surgeries	IIb	C

AS: aortic stenosis; LVEF: left ventricular ejection fraction.

**Table:** 

The choice between surgical aortic valve replacement and TAVI		
Recommendation	Grade of recommendation	Level of evidence
Surgical aortic valve replacement is recommended in patients who have indications for surgical treatment and who have low or intermediate surgical risks	I	A
In patients under consideration for TAVI and in those with high surgical risk for valve replacement, members of a Heart Team should collaborate to provide the patient with the best care possible	I	C
TAVI is recommended for patients indicated for surgical aortic valve replacement, with prohibitive surgical risk and post-TAVI life expectancy of more than 12 months	I	B
TAVI is a reasonable alternative to surgical aortic valve replacement in patients who meet indications for surgical treatment and who have high surgical risks	IIa	B
Balloon aortic valvuloplasty may be considered as a bridge to surgical or percutaneous valve replacement in severely symptomatic patients with severe aortic stenosis	IIb	C
TAVI is not recommended for patients whose existent comorbidities would impede the benefits expected from correction of aortic stenosis	III	B

TAVI: transcatheter aortic valve implantation.

### 6.4. Aortic Regurgitation

#### 6.4.1. Diagnostic Peculiarities

AR is less common in elderly patients than AS and MR.

Etiology - The most common causes of chronic AR in elderly patients are ascending aorta dilation due to SAH, primary aortic disease, calcified valve disease, and, rarely, atrioventricular block (AVB). Another cause is rheumatic cardiac disease (especially in developing countries).^232^

Symptoms - Chronic AR evolves slowly and insidiously in most cases, with very low morbidity during the asymptomatic phase. After this phase, some patients present progression of the regurgitant lesion, with subsequent LV dilation, systolic dysfunction, and, eventually, HF.^233^ Mortality rates for patients with severe AR with NYHA class II symptoms are approximately 6% yearly and almost 25% in patients in NYHA classes III or IV.^234^

Physical examination - The murmur is diastolic, decrescendo, blowing, and high frequency, and it is best heard in the left sternal border or in aortic focus. Its severity is more related to duration of murmur than to intensity. The ictus is dislocated, revealing LV volumetric overload, and its dimension is related to lesion severity. Peripheral alterations, which are characteristics of severity in young patients (increased PP, arterial neck pulsation, and systolic pulsation in the head), may be exacerbated in elderly patients, given that alterations resulting from the loss of elasticity of the great arteries may accentuate them.

Complementary exams - EKG is not very specific in AR, and the routine finding is LVO in cases with long duration. Chest radiography helps detect comorbidities, evaluate pulmonary congestion, and distinguish between acute and chronic cases. Acute cases present pulmonary congestion and normal or slightly enlarged cardiac area. Chronic cases present increased cardiac area secondary to LV dilation. Ascending aorta dilation, on the other hand, suggests that the AR is secondary to aneurysmal dilatation of the aorta. Echocardiography is the pillar of serial monitoring and evaluation of chronic AR patients. It is useful for confirming diagnosis, evaluating cause and valve morphology, estimating lesion severity, and evaluating LV dimensions, mass, and systolic function, as well as aortic root dimensions.^203^ For patients with suspected moderate or severe AR, cardiovascular magnetic resonance (CMR) provides precise quantification of regurgitant volume and fraction, in addition to precise measurements of LV volumes and function. CMR is particularly useful when the degree of LV dilatation in echocardiography seems to be greater than what would be expected. Cardiac catheterization should be performed routinely in all patients referred for surgical correction or coronary disease evaluation, or when clinical and laboratory tests are unclear or divergent regarding AR severity.^203^

#### 6.4.2. Treatment Peculiarities

In cases of severe acute AR, surgical treatment should be implemented as early as possible, especially if there are signs and symptoms of low cardiac output. In these cases, clinical treatment is inferior to surgical treatment. Inotropic drugs and vasodilators may aid clinical control while the patient is waiting for surgery.^203,235^

Clinical treatment - Clinical treatment of AR patients with vasodilators is applied to those with associated SAH and those with severe symptomatic AR and high surgical risks, especially owing to comorbidities, in order to alleviate symptoms. They are not routinely recommended for patients with mild, moderate, or severe asymptomatic chronic AR and normal systolic function.^203,235^ Studies have not demonstrated the efficacy of these drugs in slowing surgical indication in AR patients, and they do not substitute surgery when it is indicated.^236^

Surgical treatment - Patients with severe symptomatic AR, as well as some asymptomatic patients, have reduced quality of life and life expectancy without surgical treatment. Selecting the appropriate moment for and type of procedure is paramount for a satisfactory surgical result; it is, naturally, necessary to observe and respect functionality and associated comorbidities in this group of patients.^235^ Surgical treatment is indicated for patients with severe symptomatic AR or for asymptomatic patients with reduced LVEF or significant LV dilatation.^203,235^ There has recently been some speculation regarding aortic valve repair for this pathology, given that complications resulting from anticoagulant use in patients who receive mechanical prostheses are not uncommon. Scientific studies have demonstrated that valve repair is an independent predictor of better survival, with a great reduction in the need for reoperation.^237^ Few centers, however, have the experience necessary to perform this procedure, and, in elderly patients, thickened, deformed, or calcified leaflets are common findings, which complicate the procedure.^203^

Percutaneous treatment - Percutaneous aortic valve implantation is an effective option for AR patients with moderate or high risks for conventional valve replacement surgery. The use of TAVI is still off-label for AR patients, but studies have demonstrated that it is feasible and will be able to be a treatment alternative.^238^

**Table:** 

Recommendations for surgical treatment of aortic regurgitation		
Recommendation	Grade of recommendation	Level of evidence
Symptomatic patients with severe AR, regardless of LV systolic function	I	B
Asymptomatic patients, with severe AR and LVEF < 50%	I	B
Patients with severe AR scheduled to undergo other cardiac surgeries	I	C
Asymptomatic patients with severe AR, normal LV systolic function (LVEF ≥ 50%), and significant LF dilatation (LVSD > 50 mm)	IIa	B
Patients with moderate AR scheduled to undergo other cardiac surgeries	IIa	C
Asymptomatic patients with severe AR, normal LV systolic function (LVEF > 50%), progressive severe LV dilatation (LVEDD > 65 mm), and low surgical risk	IIb	C

AR: aortic regurgitation; LV: left ventricle; LVEDD: left ventricular end-diastolic diameter; LVEF: left ventricular ejection fraction; LVSD: left ventricular systolic diameter.

### 6.5. Infective Endocarditis

#### 6.5.1. Diagnostic Peculiarities

IE, which was previously prevalent in young and middle-aged patients, owing to its association with rheumatic valve disease, has progressively increased in the elderly population.^239^ In Europe and the United States, more than half of cases occur in patients > age 60. Diagnosis of IE in elderly patients may be more difficult owing to the fact that signs and symptoms such as mental confusion, fatigue, weight loss, and murmur may be attributed to age itself. The forms in which IE is present in elderly patients, such as clinical signs of stroke, HF, pneumonia, and abdominal pain, may also confuse the initial diagnosis. In some case registries, fever appears in only 2% of cases in elderly patients, in comparison with 90% of patients < age 60. Other not very specific symptoms, such as anorexia, weight loss, arthralgia, dyspnea, and headache, similarly appear in elderly patients. Classic peripheral signs of IE such as Osler's nodes, Roth spots, and petechiae, are less frequent in elderly patients, being found in 1% to 14% of cases.^240^

Laboratory and echocardiography data - hemogram may be normal or present leucocytosis, with the frequent presence of normochromic, normocytic anaemia. Erythrocyte sedimentation rate (ESR) may be elevated in 90% of cases. Positive rheumatoid factor is found in 50% of cases, and the majority of patients have proteinuria and microscopic hematuria.^241^ Blood cultures: at least 3 blood samples should be collected during the first 24 hours, with intervals of less than 15 minutes between samples, and they must be collected before beginning antibiotic therapy, given that antibiotic use is the leading cause of failure to identify the germ responsible for endocarditis. In the most developed countries, blood cultures reach 80% to 95% positivity. Echocardiogram: with the advent of echocardiography in the 1980's,^242^ the probability of diagnosing IE has increased, given that it is used to confirm the presence of vegetations, which are one of the 3 diagnostic pillars of IE, along with identification of the germ in blood culture and signs of affected valves, such as murmurs. In elderly patients, the sensitivity and specificity of transthoracic echocardiography is lower owing to the higher frequency of calcified lesions and valve prostheses, as well as the presence of obesity and thoracic deformities.^243^ TEE improves diagnostic accuracy, and it may be performed in elderly patients as safely as in younger patients.

Diagnostic criteria - In various cases of IE, diagnosis is uncertain due to the impossibility of demonstrating the existence of vegetations and to unspecific clinical manifestations, resulting in diagnostic errors. The Duke criteria, modified by Li et al.^244^ (Table 10), are the most widely used to establish IE diagnosis. Nevertheless, IE diagnosis is a difficult process, but the inclusion of clinical, laboratory, and echocardiography data reduces the chance of error.

**Table 10:** Criteria for diagnosing IE

Major criteria	
**Microbiological**	**Comments**
Typical isolated microorganism from two separate blood cultures: *Streptococcus viridans*, *Streptococcus bovis*, HACEK group, *Staphylococcus aureus*, or community-acquired enterococcal bacteremia, in the absence of a primary focus	In patients with possible IE, at least 2 blood cultures must be obtained in 2 different veins during the first 2 hours. In patients with septic shock, 3 blood cultures must be collected at 5–10 min intervals, after which point empirical antibiotic therapy should be initiated.
Or	
Persistently positive blood cultures consistent with isolated IE	
Or	
Blood culture positive for *Coxiella burnetii* or antibody titre (lgG) > 1:800 for *C. burnetii*	*C. burnetii* is not cultivated in most laboratory analyses
Evidence of endocardial involvement	
New valvular regurgitation (increases and changes in preexisting murmurs are not sufficient)	
Or	
Positive echocardiogram (TEE recommended for patients with prostheses, possible IE based on clinical criteria, or complicated IE)	Three TTE findings are considered major criteria: discrete oscillating intracardiac mass located on a valve or subvalvular structure, periannular abscess, and new dehiscence of prosthetic valve
**Minor criteria**	**Comments**
Predisposition to IE, including certain heart conditions and IV drug use	Cardiac abnormalities that are associated with IE are classified into 3 groups: ● High risk: previous IE, aortic valve disease, rheumatic valve disease, prosthetic valve, coarctation of the aorta, and complex cyanotic heart diseases ● Medium risk: mitral valve prolapse with leaflet insufficiency or thickening, isolated mitral stenosis, tricuspid valvulopathy, pulmonary stenosis, hypertrophic cardiomyopathy ● Low risk: Ostium secundum IAC, ischemic disease, previous revascularization surgery, and mitral valve prolapse without previous regurgitation, and mitral valve prolapse without regurgitation and with thin leaflets
Fever	Temperature > 38° C
Vascular phenomena	Except petechiae and hemorrhagic suffusions
	No peripheral lesions are pathognomonic of IE
Immunologic phenomena	Rheumatoid factor, glomerulonephritis, Osler nodes, Roth spots
Microbiological findings	Positive blood cultures that do not meet major criteria. Serological evidence of active infection, isolation of coagulase-negative staphylococci and organisms that rarely cause IE are excluded from this category
Cases are clinically defined as "definite IE" if they meet 2 major criteria, 1 major criterion and 3 minor criteria, or 5 minor criteria and "possible IE" if they meet 1 major criterion and 1 minor criterion or 3 minor criteria.	

HACEK: Haemophilus aphrophilus, Actinobacillus actinomycetemcomitans, Cardiobacterium hominis, Eikenella corrodens and Kingella kingae; IAC: interatrial communication; IE: infective endocarditis; IgG: immunoglobulin G; IV: intravenous; TEE: transesophageal echocardiography; TTE: transthoracic echocardiography.

#### 6.5.2. Treatment Peculiarities

As the population ages, IE affects more and more elderly individuals. More than a third of IE patients in Western countries are over age 70.^245^ Mortality in elderly patients is also higher when compared to the general population.^246^ Aging is a heterogeneous process, and it is always recommended to use AGA, which considers nutritional, functional, and cognitive status to better define prognosis as well as treatment options for this population.^247^ The majority of elderly IE patients have multimorbidities, and the most common entryways for bacteria are the digestive and urinary tracts. Furthermore, these patients have predisposing factors, such as AS, valve prostheses, and intracardiac devices.^248^ In defining treatment, the international literature makes no considerations regarding age and its consequences for treatment choice.^248-250^ AGA data and the presence of frailty syndrome are factors which should be considered in deciding on a proposed treatment.^207,240,250,251^ Table 11 shows examples of possible adaptations for elderly patients. The majority of elderly patients have decreased renal function; nephrotoxic antibiotics should, thus, be used carefully and, in some cases, even avoided in this population.^252^ Treatment of IE often entails prolonged hospital stay, which is associated with functional and cognitive decline in the elderly population. The use of outpatient parenteral antibiotic therapy should be encouraged in this population, thus avoiding the complications of prolonged hospital stay; this requires that the patient's infection be controlled and the clinical situation stabilized, in addition to long-term venous access. In the event of difficult venous access, the subcutaneous or even the oral route may be considered, depending on the antibiotic in use.^252^ Regarding surgical treatment, the indications are the same as in the general population (severe valvular lesion with HF, large vegetation with a risk of systemic embolism, and uncontrolled infection); in this context, however, AGA becomes more important in deciding on surgical treatment due to the fact that the risks of existing multimorbidities may interfere with the planned procedures. In these cases, a careful risk-benefit assessment of the procedures must be performed in an individualized manner.^253^ When surgery is indicated, the decision should be made in a multidisciplinary fashion, and, when possible, it should involve the opinion of an infectologist, cardiologist, cardiac surgeon, anesthesiologist, and geriatrician, in order to define patients who may or may not benefit from a surgical procedure with the highest possible accuracy.^252^

**Table 11:** Adaptations of the 2015 guidelines for elderly patients in accordance with comorbidities and functional status^252^

	Guidelines	Suggestion for elderly patients
Transesophageal echocardiography	Consider in all cases, in accordance with clinical suspicion	Assess risk-benefit of the procedure
Aminoglycosides	Combined to penicillin or vancomycin as first choice	Avoid, due to nephrotoxicity. Evaluate alternatives
Vancomycin	First-line treatment in beta-lactam allergic patients or in cases of MRSA	Consider daptomicine to avoid nephrotoxicity
Monitoring of antibiotic serum levels	Vancomycin and aminoglycosides	Consider also for all beta-lactam antibiotics
Intravenous therapy	All cases	Consider oral or subcutaneous route
Outpatient parenteral therapy	Only in compliant patients who have easy access to a hospital	Consider for patients for whom prolonged hospital stay may be deleterious to functional and cognitive status

MRSA: Methicillin-resistant Staphylococcus aureus. Adapted from Forestier et al., 2016.^252^

## 7. Cardiac Arrhythmias

Arrhythmias and conduction disorders are common in elderly patients, and they are an important cause of emergency room visits and hospitalization in this age group.^1^ Structural alterations in the cardiovascular system, which are promoted by aging and are associated with a higher incidence of comorbidities such as LVH, CAD, degenerative valvulopathy, SAH, LV dysfunction, and pulmonary disease, in addition to polypharmacy, are responsible for the increased prevalence of arrhythmias in this population.^254-258^ Clinical evaluation should be meticulous, as many elderly patients have atypical manifestations such as unexplained falls, intermittent mental confusion, thromboembolic events, and syncope; some are even asymptomatic and are casually detected during routine EKG.^257^ Multimorbidities, frailty syndrome, and impaired functionality and cognitive function interfere with the management of arrhythmias in this group, which should be individualized.

This section will discuss the diagnostic and treatment peculiarities of the main cardiac arrhythmias in elderly patients.

### 7.1. Syncope and Bradyarrhythmias

#### 7.1.1. Syncope and its Differential Diagnoses in Elderly Patients

Syncope has a multifactorial etiology in elderly patients. Postural hypotension, also known as orthostatic hypotension (OH) is common, secondary to medication use and severe arrhythmias. It has an average prevalence of 6%, increasing exponentially with age.^254^ It has a recurrence rate of 25% to 30% per year, in the first 2 years.^255^ It is an independent predictor of morbimortality, reduced functional capacity and institutionalization,^257^ as well as a frequent cause of hospital admission. Cardiogenic syncope has the worst prognoses, accounting for up to 20% of cases in elderly patients.^258^ Bradyarrhythmias (sinus node disease or advanced AVB) are commonly related to syncope in elderly patients. Tachyarrhythmias manifest with syncope less frequently; they are "on-off" type manifestations, with sudden onset and without short-duration prodromes. They are unrelated to orthostatic position and characterized by fast recovery. It is worthwhile to remember that AS is a possible cause of effort-induced syncope in elderly patients. The following are considered to be predictors of cardiogenic syncope, according to the Evaluation of Guidelines in Syncope Study 2 (EGSYS-2) score: EKG abnormalities, structural heart disease, palpitations before syncope, syncope during effort or in the supine position, absence of autonomic prodromes, and absence of triggering or precipitating factors (≥ 3 points suggest cardiogenic syncope).^259^ The presence of dyspnea before syncope also suggests cardiogenic etiology.^258^ Syncope due to postural hypotension is common in dehydrated patients and patients with diminished intravascular volume. Its prevalence increases with age, varying from 6% in population studies to 70% in hospitalized, institutionalized, or Parkinson's disease patients.^260^ In patients with dementia, 48% of syncope episodes occur due to OH.^261^ Syncope episodes up to 2 hours after a meal should lead to a diagnosis of postprandial hypotension. Neuromediated syncope is common in elderly patients, of which the most prevalent types are situational (associated with urination, defecation, coughing, and carotid sinus hypersensitivity).^258,259^ The presence of nausea, blurred vision, and sweating suggests a non-cardiogenic cause (OH or neurocardiogenic).^258^ Neurological syncope, due to preexisting bilateral vertebrobasilar insufficiency is often accompanied by symptoms such as vertigo and ataxia, and it has a lower prevalence. It is also necessary to consider syncope an atypical manifestation of severe diseases such as AMI, which occurs in 3% of elderly patients > age 65^262^ and is common in patients > age 85 with prevalence reaching 20%,^263^ as well as of pulmonary thromboembolism (PTE) (24% of elderly patients > age 65)^264^ and acute aortic dissection (5% to 10%).^265^

##### 7.1.1.1. Stratifying Risk of Death

The San Francisco Syncope Rules (SFS) are simple rules that evaluate risk of adverse events in syncope patients. It has 74% to 98% sensitivity and 56% specificity.^266^ The low specificity is owing to the fact that it is not very specific for cardiogenic syncope, but it makes it possible to discharge low-risk patients and hospitalize more severe cases. The following mnemonic device is used for the SFS:

C - History of CHF.

H - Hematocrit < 30%.

E - EKG abnormalities.

S - Shortness of breath.

S - SBP at admission < 90 mmHg.

(A) - Age > 75.

In a patient with syncope, any one of these findings is considered a high risk for events such as death, AMI, arrhythmia, PTE, stroke, subarachnoid hemorrhage, or emergency room re-admission and hospitalization related to a new syncope episode. When age is included, sensitivity increases to 100%, while specificity is reduced.

In conjunction with the SFS, the Short-Term Prognosis of Syncope (STePS) Study is another useful score,^267^ which evaluates the risk of events 10 days after a syncope episode. It includes only 4 independent risk factors:

- EKG abnormalities (the best predictor).
- Concomitant trauma.
- Absence of prodromes.
- Male sex.

Predictors of poor long-term (1-year) prognosis include: EKG abnormalities, ventricular arrhythmia, HF, and age > 45. The 1-year event rate (severe arrhythmia or death) varies from 0% for patients with none of the 4 risk factors to 27% in patients with ≥ 3 factors. We may, thus, consider a high risk of short-term (7 to 10 days) and long-term (1 year) events for elderly patients who have syncope and:

- Are male.
- Do not have prodromes and have syncope with concomitant trauma.
- Have dyspnea or sustained hypotension associated with the syncopal event.
- Have previous diagnosis of HF and/or ventricular arrhythmias.
- Have altered EKG at admission.

##### 7.1.1.2. General Recommendations

**Figure 2:**
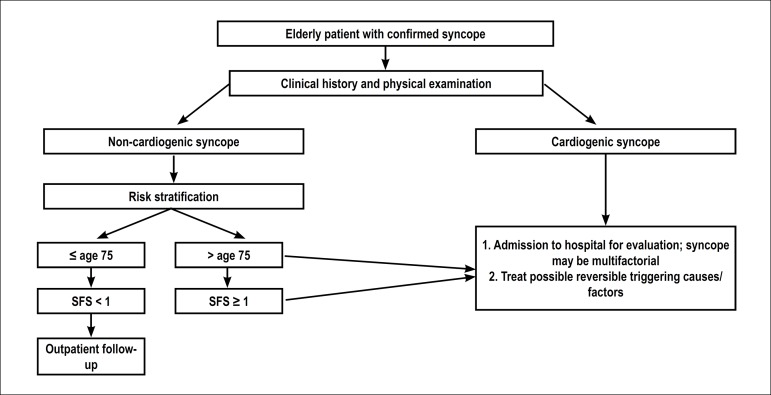
Flowchart for investigating syncope in elderly patients. SFS: San Francisco Syncope Rules.

Elderly patients with unexplained recurrent falls, which are not witnessed by third parties and which are associated with trauma, should be interpreted as possible cardiogenic syncope. Investigation should occur in a hospital environment for episodes which occurred < 1 week prior, with trauma or in patients with known heart disease. Patients with a single episode, which occurred > 1 week prior, without trauma, may be investigated as outpatients. All elderly patients > age 75 with previous heart disease diagnosis and abnormal EKG should be investigated in a hospital environment, due to the high probability of cardiogenic syncope. The flowchart in Figure 2 suggests investigation routes, based on risk stratification, clinical history, and physical examination, which will define investigation strategy and treatment.

#### 7.1.2. Diagnostic Peculiarities of Bradyarrhythmias

First-degree AVB have a prevalence of 6% to 8% in individuals ≥ age 70, and, like Mobitz I second-degree AVB, they are not predictive of cardiovascular events. Mobitz II second-degree AVB and third-degree AVB, on the other hand, have worse prognoses and require treatment. Extreme bradycardia (< 35 bpm), sinus pauses > 2 seconds, and advanced AVB are associated with structural heart disease, and they are frequently symptomatic. The association of bradycardia induced by negative chronotropic drugs, acetylcholinesterase or anticholinesterase inhibitors (rivastigmine, donepezil, and galantamine) and central alpha-blockers used for prostatic symptoms or SAH is common. Many cases are asymptomatic and are casually diagnosed during a routine check-up, especially in sedentary patients or patients with functional limitations.^268^ Common symptoms include non-rotatory dizziness, effort-induced dyspnea or fatigue, caused by chronotropic deficit. The classic "on-off" syncope (Stokes-Adams syndrome) caused by total or intermittent high-degree AVB is an alert symptom.^269^ Diagnosis may be performed via 12-derivation EKG, 24-h Holter, loop event monitor, and electrophysiological studies (EPS). Holter is indicated for bradycardia patients who have daily symptoms. Event monitors (implantable or portable) are indicated for detecting symptoms which occur rarely, but which have significant hemodynamic impairment and prolonged duration and which place the elderly patient's life at risk.^270-274^ In cases of effort-induced symptoms, treadmill ET may clarify diagnostic suspicion (chronotropic incompetence or advanced degree of AVB). EPS is indicated for patients with inconclusive 24-h Holter or loop monitor results and unexplained recurrent syncope.

**Table:** 

General recommendations for diagnosing bradyarrhythmias in elderly patients		
Recommendation	Grade of recommendation	Level of evidence
12-derivation EKG for patients with suspected bradyarrhythmia	I	C
Investigate negative chronotropic drug use and effort-induced symptoms in asymptomatic patients with bradycardia	I	C
24-h Holter for electrocardiographic correlation of symptoms with bradycardia (pre-syncope, syncope, palpitations, effort dyspnea, fatigue disproportionate to effort, or non-rotatory dizziness)	I	C
24-h Holter for patients with resting sinus bradycardia, asymptomatic patients	IIb	C
24-h Holter for patients with resting sinus bradycardia with effort symptoms to evaluate advanced degrees of block or pauses	I	C
24-h Holter for patients with high-degree AVB or total intermittent AVB, asymptomatic patients without negative chronotropic drugs	I	C
24-h Holter for patients with syncope, pre-syncope, and dizziness, whose probable cause (with the exception of bradyarrhythmias) has been identified, but whose symptoms persist in spite of treatment of the probably cause, and patients recovered from CRA	IIa	C
24-h Holter for electrocardiographic correlation of unspecific symptoms such as rotatory dizziness, dyspnea, and sweating in patients with documented bradycardia	III	C
24-h Holter for patients with dizziness	III	C
7-day Holter or loop monitor for patients with infrequent pre-syncope, syncope, palpitations, effort dyspnea, fatigue disproportionate to effort, or non-rotatory dizziness	I	C
7-day Holter or loop monitor for patients with infrequent syncope, pre-syncope, and dizziness, whose probable cause (with the exception of bradyarrhythmias) has been identified but whose symptoms persist in spite of treatment of the probably cause	IIa	C
7-day Holter or loop monitor for electrocardiographic correlation of unspecific symptoms such as rotatory dizziness, dyspnea, and sweating in patients without documented bradycardia	III	C
Treadmill ergometric test for patients with effort-induced symptoms and resting sinus bradycardia to evaluate de chronotropic incompetence	I	C
Treadmill ergometric test for patients without symptoms and resting sinus bradycardia	IIa	C
Electrophysiological study for patients with clinical suspicion of bradyarrhythmia and inconclusive non-invasive exams to measure AH intervals, HV intervals, and sinus node recovery time (investigating sinus node disease and degenerative disease of the AV node)	IIa	C

AVB: atrioventricular block; CRA: cardiorespiratory arrest; EKG: electrocardiogram.

#### 7.1.3. Treatment Peculiarities

Treating syncope - Syncope treatment in elderly patients must be multifactorial, with an approach that covers various components which may be involved in the syncopal episode. Cases of cardiogenic syncope in no way differ from the approach used in younger patients. Treatment of baseline heart disease is in accordance with specific recommendations, respecting the elderly patient's specificities.^254^ It is necessary to avoid hypovolemia and substitute vasodilatadory medications which may promote OH, by accentuating the dysautonomic response, such as beta-blockers with alpha and beta blocking action, calcium channel blockers, and central alpha-blockers. Centrally acting drugs (tricyclics, fluoxetine, aceprometazine, haloperidol, L-dopa, et al.) are also associated with risk of syncope and should be substituted.^275^ The non-pharmacological measures commonly prescribed to treat neuromediated syncope have conflicting results in the elderly population, and they also present difficulties in adherence. Not limiting sodium intake and stimulating water intake are effective, but with low adhesion.^276^ Avoiding heavy meals and meals in hot environments, as well as standing up immediately after a meal, may reduce the occurrence of postprandial hypotension. Classical medical treatment of neuromediated syncope has also not been shown to be effective in the elderly.^277^ Regarding drugs, fludrocortisone has proven efficacy in this age range, at the expense of more collateral effects, mainly edema, hypokalemia, metabolic alkalosis, weight gain, and supine hypertension.^277,278^ Treatment of cardioinhibitory syncope with pacemakers was shown to reduce syncope recurrence in a randomized clinical trial carried out in the elderly population (5% versus 61% recurrence in the pacemaker and control groups, respectively, p = 0.00000).^279^

Treating bradyarrhythmias - Treatment of bradyarrhythmias in the elderly follows the same recommendations as in younger patients.^280,281^ The suspension of negative chronotropic drugs is fundamental. In patients with symptomatic sinus bradycardia, resting HR < 40 bpm, or symptomatic pauses, indicating definitive pacemaker implant reduces symptoms and improves quality of life, but it does not interfere with prognosis.^282,283^ In patients with sinus bradycardia and dementia who need to initiate cholinesterase inhibitors, this may aggravate their bradyarrhythmia, the effect being dose dependent. Indication for a pacemaker in these patients should be individualized, as there is no evidence regarding the efficacy of this approach. In patients with advanced AVB, indication for a definitive pacemaker is associated with reduced mortality and should follow the same indications as in younger patients.^280,281^

General recommendations - With relation to treating syncope and bradyarrhythmias in the elderly, multiprofessional evaluation is important regarding the functional aspect and prognosis of comorbidities. Generally speaking, there is no specificity regarding the treatment efficacy of interventions with respect to bradyarrhythmias, and the same treatment recommendations used for younger patients should be followed. It is necessary to be attentive to non-cardiovascular use of drugs with negative chronotropic properties, as they may aggravate preexisting bradycardia.

### 7.2. Tachyarrhythmias in Elderly Patients

#### 7.2.1. Diagnostic Peculiarities

Supraventricular tachyarrhythmias (SVT) - SVT are frequent in elderly patients, and their prevalence increases with age. The most common in this age range are: atrial tachycardia, flutter, and AF.^284^ Atrial extrasystoles (AES) in patients ages 60 to 86 have an approximate prevalence of 80%, and supraventricular paroxysmal tachycardia (SVPT) has a prevalence from 10% to 15%. In individuals ≥ age 80, the prevalence of AES may reach 100%, and that of SVPT is from 25% to 30%. Effort-induced atrial arrhythmias in patients > age 80 reach a prevalence of > 10%.^285,286^ In spite of their high prevalence, SVT (with the exception of AF) are not associated with increased morbimortality.^285,286^ AES and non-sustained SVT (duration < 30 seconds) are not very symptomatic, observed with palpitation, "lightheadedness," dizziness, neck pounding, and "shortness of breath." Occasionally, dyspnea, chest pain, and syncope may occur, especially in patients with acute sustained arrhythmias, significant diastolic dysfunction, severe AS, HF, or CAD. The higher the HR, the less tolerated the arrhythmia, as a consequence of reduced cardiac output, which results in manifestations of cerebral and myocardial ischemia, arterial hypotension, and pulmonary congestion.^287^

Some arrhythmias are peculiar in elderly patients:^288,289^

a) Atrial tachycardia with AVB: presents high atrial frequency associated with slow ventricular response due to an AVB. Digitalis toxicity and hypokalemia are common causes.
b) Multifocal atrial tachycardia: is common in the presence of COPD.^285,287^ Treatment focuses on the baseline disease, considering pre-fibrillatory rhythm.
c) Accelerated junctional rhythm: digitalis toxicity and inferior wall AMI are the most common causes in elderly patients.^285,287^ Diagnosis is suggested for regular bradycardic rhythm, in the presence of AF.
d) Atrial flutter: habitually indicates structural heart disease. CAD and COPD are the most common causes in elderly patients. Elderly patients with atrial flutter have a higher chance of degeneration to AF; they are at a high risk for thromboembolic events, and they should receive a similar approach to AF cases.

Ventricular tachyarrhythmias - Ventricular extrasystoles are common in the elderly, with an incidence of 70% to 90%.^284,287,288^ They do not generally produce symptoms, unless they are very frequent. Symptomology is variable; patients may perceive repetitive heart beats or the sensation that their "heart is going to stop," due to compensatory pauses. They are associated with risk of death in the presence of structural heart diseases. Treating arrhythmia in an isolated manner, however, does not reduce risk in elderly patients with CAD.^286,289^ Pre-syncope, syncope, low output, pulmonary congestion, behavior disorder, and disorientation are frequent clinical manifestations of poor prognosis. Ventricular tachycardia (VT) is frequently associated with structural heart disease. LVH is an important determinant of ventricular arrhythmia,^287^ as well as HF, which increases the incidence of VT from 2% to 4%, in patients without HF, to 20% to 80%.^287^ In these patients, the presence of complex ventricular arrhythmia is associated with an increase in total mortality, cardiac mortality, and sudden death. The worse the ventricular dysfunction, the more complex and severe the ventricular arrhythmia will be. Thus, patients with LV dysfunction or LVH with complex ventricular arrhythmia should be considered at a high risk of sudden death, even if they are asymptomatic. In elderly patients without heart disease, the finding of tachyarrhythmias on Holter has no prognostic implications.^286^

Based on these premises, with respect to diagnostic evaluation of tachyarrhythmias in elderly patients, these Guidelines recommend:

**Table:** 

Recommendation	Grade of recommendation	Level of evidence
Inquiry about all medications in use and risk analysis of induced arrhythmias or prolonged QT	I	C
12-derivation EKG in all patients at each clinical visit, even in the absence of symptoms	I	C
Calculation of QTc interval for all patients who report palpitation	I	C
Calculation of QT interval for all patients with polymorphic VT	I	B
24-h Holter to evaluate symptoms of palpitation, syncope, and unexplained falls	I	B
24-h Holter for asymptomatic patients with normal LV function and EKG with LVH	IIa	B
24-h Holter for asymptomatic patients with depressed LV function and EKG with LVH	I	A
24-h Holter for patients recovered from VF/VT before hospital discharge	IIa	C
24-h Holter for patients recovered from VF/VT during outpatient follow-up to evaluate therapy efficacy	IIb	C
24-h Holter for asymptomatic patients with simple ventricular arrhythmia during the initial exam, with normal LV function and EKG with LVH, during outpatient follow-up to evaluate therapy efficacy	III	C
24-h Holter for asymptomatic patients with complex ventricular arrhythmia during the initial exam, with normal LV function and EKG with LVH, during outpatient follow-up to evaluate therapy efficacy	IIb	C
24-h Holter for asymptomatic patients with simple ventricular arrhythmia during the initial exam, with depressed LV function and EKG with LVH, during outpatient follow-up to evaluate therapy efficacy	III	C
24-h Holter for asymptomatic patients with complex ventricular arrhythmia during the initial exam, with depressed LV function and EKG with LVH, during outpatient follow-up to evaluate therapy efficacy	IIa	C
24-h Holter for asymptomatic patients with normal LV function and EKG	III	B
Ergometric test in patients without contraindications who have effort-induced palpitations	I	C
Ergometric test in patients without contraindications who have palpitations associated with chest angina	I	C
Ergometric test in patients without contraindications who have resting palpitations	III	C
Ergometric test in asymptomatic patients without contraindications to investigate arrhythmia	III	C
Echocardiogram in all patients with palpitations	IIb	B
Echocardiogram in patients with LVH on EKG, asymptomatic patients	IIa	B
Echocardiogram in patients with palpitation and dyspnea	I	B
Echocardiogram in patients with LVH and cardiac murmur, asymptomatic patients	I	B
Investigation of ischemic etiology in all patients with supraventricular tachycardia	III	C
Investigation of ischemic etiology in all patients with supraventricular tachycardia and angina	I	C
Investigation of ischemic etiology in all patients with complex ventricular tachycardia	I	C
Magnetic resonance in patients with complex ventricular arrhythmia, whose other exam results are normal, to investigate arrhythmogenic RV dysplasia, myocardial fibrosis, and asymmetric apical hypertrophy	I	C
Magnetic resonance in all patients with VT	III	C
Magnetic resonance in all patients with SVT	III	C
EPS in patients with high SD risks (unexplained syncope and complex ventricular arrhythmia on Holter or trifascicular block, in order to clarify syncope etiology)	I	C

EKG: electrocardiogram; EPS: electrophysiological study; LV: left ventricle; LVH: left ventricular hypertrophy; RV: right ventricle; SD: sudden death; SVT: supraventricular tachyarrhythmia; VF: ventricular fibrillation; VT: ventricular tachycardia.

#### 7.2.2. Treatment Peculiarities

Treatment principles for tachyarrhythmias in the elderly are similar to those in younger patients; however, treatment is more frequently influenced by the presence of baseline heart diseases such as CAD, LV dysfunction, LVH, and comorbidities such as chronic renal insufficiency (CRI) and COPD.^290^ Non-sustained atrial arrhythmias (supraventricular extrasystoles and atrial tachycardias), generally, do not require treatment. In most cases, they are associated with baseline respiratory diseases, whose treatment, associated with avoiding stimulants such as caffeine, cigarettes, soft drinks, black tea, and fast-acting beta-agonist drugs, is normally sufficient to reduce the number of events and symptoms. Otherwise, the use of calcium channel blockers in patients with COPD (contraindicated in cases of LV dysfunction) or beta-blockers (in low doses and selectively, such as bisoprolol or metoprolol), in patients without contraindication, may be indicated. SVPT is usually caused by reentrant mechanisms, and may be interrupted by vagal maneuvers, such as the Valsalva maneuver, coughing, and vomiting. Due to the risk of arterial embolism, carotid sinus massage should be avoided in all elderly patients unless the presence of significant carotid disease has been excluded. If the attempted vagal maneuvers do not succeed in reversing arrhythmia, chemical cardioversion should be attempted. The first-choice drug should initially be adenosine, with electrocardiographic monitoring. Second-line drugs are calcium channel blockers (verapamil, diltiazem), if LV function is normal, and beta-blockers, in the presence of CAD. Digoxin should be restricted to patients with depressed LV function. In cases that do not respond to first- and second-line agents, class III antiarrhythmic drugs (amiodarone or sotalol) should be used. Beta-blockers and calcium channel blockers are equally effective in maintaining SR and avoiding recurrence of arrhythmia^290^ (Table 12). In the event of hypotension, signs of low cerebral blood flow, pulmonary congestion, or chest angina, electric cardioversion should be performed at 50 to 75 J. Catheter ablation for treating sustained SVPT whose mechanism is nodal reentrant or an accessory pathway is as effective in elderly patients as it is in younger patients, with a success rate of > 95%.^291-295^ Elderly patients have a higher risk of complications such as perforation, vascular lesion, renal insufficiency, a higher tendency to develop AF, and thromboembolic events after the procedure. Nevertheless, larger complications occur in < 3% of elderly patients.^292,293^ It should be considered the treatment of choice for patients with frequent episodes (> 2 events/year, in spite of medical treatment) or patients with contraindications to the previously cited drugs, such as sinus bradycardia, hypotension, broncospasms, and severe LV dysfunction, as well as for patients who do not wish to undergo medical treatment.

**Table 12:** Drugs used to treat SVPT in elderly patients^290^

Cardioversion in the emergency room					
	**Drug**	**Initial dose**	**Repeat**	**Total dose**	**Precautions**
1^st^ choice	Adenosine	6 mg in rapid bolus IV over 10 seconds	12 mg every 15 minutes	30 mg	Patients with CAD and active bronchial asthma
2^nd^ choice	Verapamil	5 mg IV over 3 to 5 minutes	5 mg after 15 minutes	10 mg	LV dysfunction and hypotension
Patients with severe LV dysfunction	Amiodarone	300 mg IV over 30 minutes diluted in 0.9% saline solution or 100 to 250 mL 5% glucose solution	-	300 mg in bolus IV and 900 to 1,200 mg over the following 24 h	May be associated with digitalis IV to better control HR
**Drugs used for maintenance following reversion to sinus rhythm **					
Calcium channel blockers	Diltiazem (start with short half-life formulations and, if tolerated, substitute with extended release formulations, following dose adjustment)	30 mg 3×/day	Increase the dose by 50% every 14 days, if well tolerated, until the desired resting HR (60 to 70 bpm) has been reached	180 to 240 mg/day	Use caution with tachycardia-bradycardia syndrome and LV dysfunction
	Verapamil	120 mg/day	Idem	240 mg/day	Idem
Beta-blockers	Metoprolol	50 mg/day		Double the dose until the desired HR of 60 to 70 bpm has been reached	200 mg/day
	Atenolol	25 mg/day			200 mg/day
	Propranolol (In this order of preference, on account of liposolubility)	40 mg/day			240 mg/day
	Carvedilol	3.125 mg 2×/day		Double the dose every 2 weeks	25 mg 2×/day
Digoxin	Preferential in patients with HF	0.125 mg/day	Take care with patients > age 75 and creatinine > 1.5 mg/dL		0.25 mg/day (In the most elderly patients, debilitated patients, and patients with ERD, the dose should be adjusted in accordance with response and maintained at lower doses to 0.125 mg, 2–3×/week)
Amiodarone	Pay attention to collateral effects, especially those that are thyroid-related	600 mg/day for 10 days	Reduce to 400 mg/day for 10 days and maintain 200 mg/day	Monitor hepatic function, thyroid function, QTc interval, and eye fundus every 6 months	Maintain 100 to 200 mg/day

DAC: doença arterial coronariana; FC: frequência cardíaca; IC: insuficiência cardíaca; IRC: insuficiência renal terminal; IV: via intravenosa; TPSV: taquicardia paroxística supraventricular; VE: ventrículo esquerdo.

General recommendations - Treatment of tachyarrhythmias in elderly patients: treatment of tachyarrhythmias in elderly patients, especially those between the ages of 65 and 75, should be similar to that in younger patients. In patients > age 75, individualization of conduct is recommended with multiprofessional evaluation that takes into consideration not only age, but also comorbidities, cognitive function, functional capacity, patient preferences, and severity of symptoms.^296,297^

### 7.3. Atrial Fibrillation

#### 7.3.1. Diagnostic Peculiarities

AF is the most common persistent arrhythmia in elderly patients.^298^ Its prevalence and incidence double every decade after age 60, affecting as many as 8% to 10% of patients > age 80 and 27% of patients > age 90.^287-301^ It may occur isolatedly as a consequence of morphological and electrophysiological alterations inherent in aging of the atrial myocardium and sinus node, known as "isolated AF" or "lone atrial fibrillation." Truly isolated AF is, however, rare in elderly patients.^302^ In general, it is associated with structural heart diseases: CAD, SAH, mitral valvulopathy, and HF.^303^ Subclinical hyperthyroidism triples the risk of AF.^300^ Patients with clinical hyperthyroidism may present episodes of paroxysmal AF. Other causes of AF in elderly patients include: obstructive sleep apnea-hypopnea syndrome (commonly called paroxysmal AF),^303^ sinus node disease, and dilated cardiomyopathy, which are generally associated with AF with low ventricular response. Special attention should be paid to sinus node disease represented by tachycardia-bradycardia syndrome, where recurring paroxysmal AF is observed with a sudden stop followed by a long or asystolic pause, which is a frequent cause of unexplained syncope in the elderly. After adjusting for coexisting CVD, mortality in patients with AF is 1.5 to 1.9 times higher that in patients of the same age without AF.^299^ This higher rate of mortality is mainly due to the 4- to 5-fold increase in the occurrence of stroke, a risk which proportionally increases after age 50 (< 1.5% in patients < age 50 and approximately 23.5% in patients > age 80).^304,305^ Diagnosis of AF in elderly patients is initially made by physical examination, anamnesis, and EKG. As many as 20% of AF diagnoses in elderly patients occur casually, during clinical visits, in patients without complaints, especially in cases of permanent AF and ventricular response < 100 bpm, which occurs on account of concomitant AV nodal disease or use of beta-blockers.^287^ The most frequent symptoms in elderly patients are: dyspnea, asthenia, dizziness, easy fatigue, decreased tolerance to exercise, sweating, polyuria, syncope, and palpitation. Permanent AF is related to silent thromboembolic events which, associated with chronic decreased cerebral blood flow and cerebrovascular alterations inherent in aging, are responsible for cognitive and motor impairments, such as slowing, motor incoordination, and dementia, which are initially discrete, but progressive and which may go unnoticed and delay diagnosis.^306^

**Table:** 

General recommendations regarding AF diagnosis in the elderly		
Recommendation	Grade of recommendation	Level of evidence
Inquiry about all medications in use and risk analysis of induced arrhythmias or prolonged QT	I	C
12-derivation EKG in all patients with irregular rhythm to diagnose AF, even in the absence of symptoms	I	C
12-derivation EKG in all patients with diagnosis of AF, at each clinical visit	IIb	C
24-h Holter for evaluation of HR control	IIa	B
24-h Holter as follow-up, after rhythm control, in asymptomatic patients	IIa	C
24-h Holter for patients who complain of palpitations and for those with sinus rhythm following rhythm control	I	C
24-h Holter for patients with sinus rhythm, after stroke, to investigate paroxysmal AF	I	C
Transthoracic echocardiography in all patients with AF, with no prior diagnosis of CHF	I	C
Transthoracic echocardiography in all patients with AF	IIa	C
Transesophageal echocardiography in patients with AF > 48 h, for reversion to SR	I	C
Transesophageal echocardiography in patients with AF, after stroke, to investigate emboligenic focus	IIb	C

AF: atrial fibrillation; CHF: congestive heart failure; EKG: electrocardiogram; HR: heart rate; SR: sinus rhythm.

#### 7.3.2. Treatment Peculiarities

Treatment of AF in elderly patients does not differ from that in younger patients. Oral anticoagulation (unless contraindicated) and the elimination of precipitating or reversible factors that induce paroxysmal AF or loss of ventricular frequency control in patients with persistent or permanent AF are the bases of AF treatment in elderly patients.^307,308^ The decision to control HR or SR should be individualized; however, as an initial routine strategy, rhythm control has no benefits over HR control in asymptomatic patients in this age range.^309,310^

##### 7.3.2.1. Heart Rate Control

Lenient strategies for HR control (target baseline HR < 110 bpm) is as effective for controlling symptoms as restrictive HR control (target resting HR < 80 bpm), except in cases of ventricular dysfunction, where caution is necessary to avoid significant bradycardias (HR < 50 bpm).^311,312^ Beta-blockers, used alone, manage to adequately control HR in 42% of elderly patients,^312^ and they should be the first-choice drug for this purpose. Combination with non-dihydropyridine calcium channel blockers should be used cautiously and only in patients without LV dysfunction. Attention should be paid to the condition worsening or to constipation appearing with their use, notably with verapamil, in addition to bradycardia and inferior member edema. Digoxin is less effective when used alone for controlling HR during effort. It is an acceptable choice for physically inactive patients, patients > age 80, and patients in whom other treatments have been ineffective or are contraindicated, and it should be used with due caution.^311,313^ In cases of tachycardia-bradycardia syndrome and in patients who do not tolerate pharmacological HR control, pacemaker implant or atrioventricular node ablation followed by pacemaker implant may be indicated.^314,315^ Rhythm control should be reserved for specific circumstances, particularly when symptoms cannot be contained by HR control, given that it is related to a higher number of hospitalizations due to the collateral effects of antiarrhythmic drugs (AAD) and the complications of invasive procedures, mainly in persistent AF with long duration. The strategy of rhythm control does not dispense with anticoagulation.^316^ Control may be via AAD, electric cardioversion, or interventional procedures. Electric cardioversion restores SR and is indicated for acute cases of AF that do not respond to pharmacological therapy and that have hemodynamic instability. The basis for choosing AAD either for chemical cardioversion or for maintenance of rhythm depends on the baseline heart disease and the comorbidities, taking the occurrence of major collateral effects into consideration, due to decreased physiological function and the interactions between multiple medications common in elderly patients. Propafenone, sotalol, and amiodarone may be used for patients with minimal or no structural heart disease, bearing in mind that there is a higher risk of collateral and proarrhythmic effects in elderly patients when using propafenone and sotalol. For patients with structural heart disease (LVH with interventricular septum > 12 mm or coronary disease), sotalol or amiodarone are indicated. Amiodarone is reserved for elderly patients with reduced HF and LVEF.^317^ Catheter ablation may be useful in healthy elderly patients who are symptomatic, without many comorbidities, without underlying heart disease, with AF paroxysms, and patients who are refractory to treatment or patients who do not wish to use AAD and who have no renal dysfunction. This procedure should be performed in a center with a great deal of experience.^318^

#### 7.3.3. Oral Anticoagulants in Elderly Atrial Fibrillation Patients

The most feared complication in AF is thromboembolic events, notably stroke, whose incidence and severity increase with age.^319^ It is the cause of up to 25% of strokes in elderly patients.^320^ Oral anticoagulant therapy reduces the risk of stroke in elderly patients with non-valvular AF by 64%. It is thus superior to aspirin, which reduces the risk by only 22%, and is no longer recommended for stroke prevention in AF patients.^319,320^ Double antiplatelet aggregation has not demonstrated benefits for preventing thromboembolic events in patients with AF and is not recommended.^321^ The risk of thromboembolism in AF may be calculated using risk factor scores.^322^ For evaluation of thromboembolic risk, the congestive heart failure, hypertension, age ≥ 75, diabetes mellitus, prior stroke, or transient ischemic attack (CHADS_2_) score has been the most used. Its variables are age (≥ 75) and the presence of comorbidities (HF, SAH, diabetes mellitus, and previous history of thromboembolism). Thromboembolism is worth 2 points, and the other variables are worth 1. Anticoagulation is indicated for patients with scores ≥ 2, as they are at a high risk of events.^323^ In 2010, the CHA_2_DS_2_-VASc score was proposed, considering a higher risk for female patients over age 65 and patients with peripheral arterial disease (1 point for the following variables: HF, hypertension, age between 65 and 74, diabetes mellitus, and peripheral arterial disease; 2 points for age 75 or over and previous thromboembolic event), resulting in higher scores for more elderly patients, women, and peripheral arterial disease patients. These Guidelines, following the recommendations of the most recent guidelines^324,325^ for treating AF, recommend the use of the CHA_2_DS_2_-VASc clinical score for defining start of anticoagulation in men with scores of 2 or more and women with score of 3 or more. In low-risk patients (men with scores of 0 and women with scores of 1), we recommend the use of echocardiography parameters, such as increased LA and auricular flow velocity, the presence of moderate to accentuated spontaneous contrast, or LA/auricular thrombus as an additional stratification for CHA_2_DS_2_-VASc. If a patient presents any one of these findings, anticoagulation is indicated.^326,327^ After defining the risk of a thromboembolic event, it becomes necessary to stratify the risk of bleeding, before beginning anticoagulant therapy. The most used risk score for bleeding during anticoagulation is the HAS-BLED, where a score > 3 indicates a high risk of hemorrhage due to oral anticoagulants and includes, in addition to age range (> 65), variables such as SAH with SBP > 160 mmHg (1 point), renal or hepatic dysfunction (1 point each), prior history of stroke (1 point), bleeding (1 point), labile INR (1 point), and drug or alcohol use (1 point each).^328^ Data on the isolated influence of age on the risk of bleeding are conflicting; for this reason, age should not be used to contraindicate anticoagulation.^329^ Vitamin K antagonists, especially warfarin, are the pillar of oral anticoagulation in patients with AF, significantly reducing stroke and mortality attributed to AF.^330^ Variability of INR with warfarin use depends not only on the dose used, but also on other medications and certain types of foods.^331^ In an observational study, labile INR has been described in 21.3% of patients ages 40 to 89,^328,329^ according to which the risk of INR ≥ 5 increases by 15% with each 10-year increment. As a result of this greater risk, it is necessary to monitor INR in elderly patients (especially those > age 75) more regularly and at more frequent intervals (grade of recommendation I, level of evidence B). These Guidelines recommend the use of low initial doses for elderly patients < age 85 (3 to 4 mg) and 2.5 mg for elderly patients ≥ age 85, patients with frailty syndrome, malnutrition, or hepatic disease and moderate to advanced renal insufficiency (creatinine clearance < 30 mL/min). The INR is at 3 days, with a new dose at 7 days, if there is dose adjustment, and 14 days, if the dose remains stable. It is weekly during the first 90 days in patients with greater risks, whatever they may be, > age 85, frailty, hepatic or renal insufficiency, history of falls, cognitive impairment, low level of education, and initial treatment. In other patients, evaluation of INR may occur every 15 days during the first 90 days of treatment, and may be monthly afterwards, in cases with stable INR. Oral anticoagulation with warfarin is, thus, safe in elderly patients, provided that precautions in indication and follow-up are respected. Warfarin is the least expensive oral anticoagulant, and its antagonist (vitamin K) is widely available to reverse the drug's anticoagulant effect.

Recently, non-vitamin K antagonist oral anticoagulants have become available with the advantages of not requiring constant monitoring of blood coagulation and presenting fewer drug interactions. They include direct thrombin inhibitors (dabigatran) and direct inhibitors of factor Xa (rivaroxaban, apixaban, and edoxaban). A meta-analysis of the main randomized clinical trials with non-vitamin K antagonist oral anticoagulants^330^ has shown a significantly lower risk of stroke or systemic embolism compared with warfarin (relative risk [RR] = 0.81, 95% confidence interval [95% CI] = 0.73 to 0.91), as well as a lower risk of intracranial bleeding (RR = 0.48, 95% CI = 0.39 to 0.59), but not of major bleeding (RR = 0.86, HF 95% = 0.73 to 1.00). Findings were similar to those described in a second meta-analysis^331^ with participants ≥ age 75. Notwithstanding the clear benefit of non-vitamin K antagonist oral anticoagulants, as well as the fact that they are safer regarding intracranial bleeding, this complication has relatively low rates (< 1%/year) even with warfarin (0.76% to 0.85% with warfarin and 0.26% to 0.49% with non-vitamin K antagonists).^331^ The new oral anticoagulants are, thus, the safest option for anticoagulation in elderly patients with higher risks of bleeding, patients with difficulties in adhering to INR monitoring, patients using multiple medications, or patients who individually opt for them. It is, nonetheless, necessary to adjust doses according to renal function and age (< or > 75)^330,331^ (grade of recommendation I, level of evidence B). Until recently, there were some concerns due to the lack of a specific antidote for reversing the anticoagulant effects of non-vitamin K antagonists; idarucizumab, however, has been introduced and was recently approved for use in humans in order to reverse the effects of dabigatran.^332^

**Table:** 

Recommended doses for elderly patients				
	Dabigatran	Rivaroxaban	Apixaban	Endoxaban
Commercial presentation	150 mg 110 mg	20 mg 15 mg 10 mg	5 mg 2.5 mg	30 mg 60 mg
Dose	150 mg CrCl > 50 ml/min	20 mg CrCl > 50 ml/min	5 mg CrCl > 30 ml/min	60 mg CrCl > 50 ml/min
	110 mg CrCl between 30 and 50 ml/min	15 mg CrCl between 30 and 50 ml/min	2.5 mg CrCl 15-30 ml/min or Two of the following criteria: ≥ 80 years old Weight ≤ 60 kg Creatinine ≥ 1.5 mg	30 mg CrCl 15-50 ml/min or weight ≤ 60 kg
Posology	2 x day	1 x day	2 x day	1 x day
Particularities	Dyspepsia is common	Higher risk of GI bleeding than warfarin		
	Avoid if CrCl < 30 ml/min), recent stroke, and severe active hepatic disease	Avoid if CrCl < 15 ml/min) and severe active hepatic disease	Avoid if CrCl < 15 ml/min) or Creatinine > 2.5 mg and severe active hepatic disease	Avoid if CrCl < 15 ml/min or severe hepatic disease

CrCl: creatinine clearance; GI: gastrointestinal. Source: European Heart Journal.^324^

##### 7.3.3.1. General Recommendations

1. Unless there are formal contraindications to anticoagulation, elderly AF patients should begin anticoagulation, if their CHADS_2_VCAS_2_ scores are ≥ 2 for men and ≥ 3 for women (grade of recommendation I, level of evidence A).^324,325^ If the CHADS_2_VCAS_2_ score is < 2 for men or < 3 for women and LA size is > 5.0 cm (or area indexed by body surface > 30 mm/m^2^) on transthoracic echocardiography, anticoagulation should also be initiated (grade of recommendation IIa, level of evidence B). Elderly patients < age 65, with CHADS_2_VCAS_2_ = 0 for men or 1 for women should only start anticoagulation if LA > 5.0 cm or in the presence of moderate to severe spontaneous contrast or thrombus on transesophageal echocardiography (grade of recommendation IIa, level of evidence B).
2. The HAS-BLED score is recommended to evaluate risk of bleeding during anticoagulation (grade of recommendation I, level of evidence B). Elderly patients are considered at higher risks if they are > age 85, are fragile, have renal or hepatic insufficiency, have moderate to severe cognitive impairment, or have low levels of education, as well as during the first 90 days of treatment with anticoagulants. In these patients, anticoagulation is recommended with dose adjustment and more regular follow-up; however, it should not be contraindicated (grade of recommendation I, level of evidence C).
3. In parallel, the risk of hemorrhagic complications may further be reduced by controlling SAH and the risk of falls, as well as by paying attention to the introduction of new drugs in association with antiplatelet medications and antibiotics, which may interfere with serum levels or increase the risk of bleeding.
4. For warfarin patients, INR is recommended 5 to 7 days after beginning antibiotic therapy (grade of recommendation I, level of evidence C).
5. Regarding the choice of anticoagulant, current evidence demonstrates that direct oral anticoagulants (DOAC) are preferable to warfarin, except in patients with moderate to severe mitral stenosis and patients with valve prostheses (grade of recommendation I, level of evidence A).^324,325^ These Guidelines, however, also recommend warfarin use, in situations of availability or preference, owing to the fact that it is an oral drug that is well known, inexpensive, and widely available to patients through the public system in Brazil, as well as to the fact that it has an antagonist (vitamin K) available to reverse its anticoagulant effect (grade of recommendation I, level of evidence A).
6. DOAC are a safe option for anticoagulation in elderly patients with higher risks of bleeding, in patients with difficulties in adhering to INR monitoring, patients using multiple medications, or patients who individually opt for them. It is, nonetheless, necessary to adjust doses according to renal function and age^330,331^ (grade of recommendation I, level of evidence A). Rivaroxaban and edoxaban are the DOAC of choice given their use practicality (taken once a day). In patients with dyspepsia, dabigatran should be avoided (grade of recommendation I, level of evidence B). No DOAC have been tested with severe renal insufficiency.^324^ For this reason, these Guidelines do not recommend using them in patients with creatinine clearance < 30 ml/min, in which case warfarin is preferable (grade of recommendation I, level of evidence B).
